# Combining Plant Bioactives With Antibiotics for Enhanced Antibiofilm Activity Against Uropathogenic *Staphylococcus* spp. and Cytotoxicity Evaluation

**DOI:** 10.1155/adpp/7461209

**Published:** 2025-08-04

**Authors:** Ulrich Joël Tsopmene, Christian Ramsès Kuate Tokam, Larissa Yetendje Chimi, Nathalie Boulens, Eric Allémann, Florence Delie, Clautilde Teugwa Mofor, Jean Paul Dzoyem

**Affiliations:** ^1^Department of Biochemistry, Faculty of Science, University of Dschang, Dschang, Cameroon; ^2^Department of Pharmaceutical Sciences, Faculty of Medicine and Pharmaceutical Sciences, University of Dschang, Dschang, Cameroon; ^3^School of Pharmaceutical Sciences, University of Geneva, Geneva, Switzerland; ^4^Institute of Pharmaceutical Sciences of Western Switzerland, University of Geneva, Geneva, Switzerland; ^5^Department of Biochemistry, Faculty of Science, Laboratory of Phytobiochemistry and Medicinal Plants Studies, University of Yaoundé I, P.O. Box 812, Yaoundé, Cameroon

## Abstract

Urinary tract infections (UTIs) are one of the most important causes of morbidity and healthcare spending. Combination therapy is the treatment of choice for biofilm-associated infections due to the simultaneous action of two drugs on two separate cellular targets and their safety. This study aimed to evaluate the effect of the combination of some bioactive natural products with conventional antibiotics against the biofilm of uropathogenic *Staphylococcus* spp. Antibacterial and antibiofilm activities were determined by the broth microdilution test. The checkerboard method was used for combination studies. The cytotoxicity of the best synergistic combinations was evaluated on Raw 264.7 macrophage cells and urinary epithelial cells (UROtsa) using the 3-(4,5-dimethylthiazol-2-yl)-2,5-diphenyltetrazolium bromide (MTT) assay. Plumbagin also showed the best biofilm-inhibiting and eradicating activities compared to curcumin, berberine, thymol, quercetin, and gallic acid. The best synergistic combinations against biofilm inhibition and eradication were C1: cefixime (5.33 µg/mL) + thymol (32 µg/mL); C2: cefazolin (1.16 µg/mL) + thymol (21.33 µg/mL); C3: amikacin (0.18 µg/mL) + curcumin (37.33 µg/mL); C4: kanamycin (0.25 µg/mL) + curcumin (14 µg/mL); and C5: amoxicillin (1.16 µg/mL) + curcumin (21.33 µg/mL). Time-kill studies revealed that the highest antibiofilm activities of the best combinations were observed at 24 h. Eradication activities were more significant than inhibitory activities. Compared to C3, C4, and C5 combinations, C1 and C2 combinations showed less cytotoxicity against the two tested cell lines UROtsa and Raw 264.7. This study shows that the best antibiofilm synergistic effect was obtained with the combination of thymol with cefixime and cefazolin, associated with low cytotoxicity. These associations could be considered potential candidates for the development of combination therapies against *Staphylococcus* spp. biofilm-associated infections. While this study demonstrates promising *in vitro* results, further *in vivo* validation is necessary to confirm the efficacy and safety. Additionally, mechanistic studies are needed to understand the synergistic pathways, and future research should address scalability and formulation for clinical use.

## 1. Introduction

Urinary tract infections (UTIs) are among the most prevalent infectious diseases globally, affecting millions of individuals annually and constituting a significant public health concern [1]. Among the wide range of pathogens responsible for UTIs, *Staphylococcus* species, including *Staphylococcus aureus*, *Staphylococcus saprophyticus*, and *Staphylococcus epidermidis*, have emerged as significant uropathogens [2–4]. These bacteria are capable of causing both uncomplicated and complicated UTIs, with the latter often associated with biofilm formation and multidrug-resistant strains [5]. The growing resistance of uropathogenic *Staphylococcus* spp. to conventional antibiotics (ATBs) necessitates innovative strategies to combat these infections, particularly approaches that target biofilm-associated resistance mechanisms. The urgent need to address biofilm-related infections has led to the exploration of alternative therapeutic options, including the use of bioactive plant natural products (NPs) and recent Food and Drug Administration (FDA)–approved ATBs like ceftobiprole medocaril (Zevtera) for methicillin-resistant *Staphylococcus aureus* (MRSA) infections. These developments underscore the importance of innovative strategies, such as combination therapies, to combat resistance [6, 7].

Biofilms are structured microbial communities encased in a self-produced extracellular polymeric matrix, which offers protection against environmental stresses, including ATB treatment and host immune responses [8]. In the context of UTIs, *Staphylococcus* spp. frequently form biofilms on urinary catheters, bladder epithelium, and other urinary tract surfaces, contributing to persistent infections and treatment failures [9]. Biofilm-associated cells exhibit up to 1000-fold higher resistance to ATBs compared to their planktonic counterparts, making eradication particularly challenging [10]. The ability of uropathogenic *Staphylococcus* spp. to establish biofilms is facilitated by an array of virulence factors, including adhesins, autolysins, and extracellular enzymes [11]. *S. saprophyticus*, for instance, is a leading cause of uncomplicated UTIs in young women, while *S. aureus* is associated with complicated UTIs and can lead to severe outcomes such as bacteremia and pyelonephritis [12, 13]. The urgent need to address biofilm-related infections has led to the exploration of alternative therapeutic options, including the use of bioactive plant NPs.

NPs derived from plants have long been recognized for their therapeutic potential, serving as sources of novel antimicrobial compounds [14]. Many plant-derived secondary metabolites, such as flavonoids, alkaloids, phenolics, and terpenoids, exhibit significant antimicrobial activity, including effects against biofilm-forming pathogens [15]. These compounds can disrupt biofilm formation by inhibiting quorum sensing, reducing extracellular polymeric substance production, or directly killing bacterial cells within the biofilm [16]. Some bioactive plant NPs with demonstrated antibiofilm activity include curcumin from *Curcuma longa* [17], eugenol from *Syzygium aromaticum* [18], and resveratrol from grapes (*Vitis vinifera*) [19]. These compounds not only inhibit biofilm formation but also enhance the efficacy of conventional ATBs by sensitizing bacteria to these drugs [20]. The ability of plant NPs to target multiple pathways involved in biofilm development makes them attractive candidates for combination therapy.

The concept of combining bioactive plant NPs with conventional ATBs has gained attention as a strategy to enhance antimicrobial efficacy and combat drug resistance [21]. Synergy occurs when the combined effect of two agents is greater than the sum of their individual effects. Several studies have reported synergistic interactions between plant-derived compounds and ATBs, resulting in enhanced antimicrobial activity, reduced effective doses, and decreased risk of resistance development [22]. For instance, studies have shown that the combination of epigallocatechin gallate from green tea with β-lactam ATBs significantly enhances the killing of MRSA [23]. Similarly, berberine, an isoquinoline alkaloid, has been shown to synergize with aminoglycosides against *Staphylococcus* spp. by disrupting membrane integrity and increasing ATB uptake [24]. We previously reported a synergistic effect of the combination of thymol and piperine with aminoglycoside ATBs against *Klebsiella pneumoniae* and *Salmonella enterica* serovar-resistant strains and biofilm-associated infections [25, 26]. Similarly, we also reported a synergistic effect of the combination of sinapic acid, thymol, and curcumin with ATBs against *Pseudomonas aeruginosa* biofilm inhibition and virulence-associated factors [27]. These findings highlight the potential of plant NPs to restore the efficacy of ATBs against biofilm-forming pathogens.

While the antimicrobial and antibiofilm activities of plant NPs are well documented, their cytotoxicity and safety profiles must also be carefully evaluated. Some plant-derived compounds exhibit dose-dependent cytotoxic effects on mammalian cells, which may limit their therapeutic applicability [28]. Therefore, the selection of NPs for combination therapy should prioritize compounds with high antimicrobial efficacy and low toxicity. Such approaches may facilitate the safe and effective use of plant-derived compounds in combination therapies.

Based on their wide range of pharmacological activity, six bioactive plant natural compounds were selected for this study. They included thymol, a monoterpene of essential oil found in *Thymus vulgaris* with antibacterial and antifungal activities [29]; curcumin, a coumarin compound found in *Curcuma longa* possessing various biological activities [30]; berberine, an isoquinolone alkaloid isolated from *Berberis vulgaris* having antibacterial properties [31]; quercetin, a flavonoid compound present in various plants [32]; gallic acid, a 3,4,5-trihydroxybenzoic acid found in many plants such as Anacardiaceae, Fabaceae, and Myrtaceae [33]; and plumbagin, a naphthoquinone obtained from the root of *Plumbago zeylanica* [34]. Although these compounds were previously investigated individually for their activity against *S. aureus*, studies reporting their activity against *S. saprophyticus* and *S. epidermidis* uropathogens are very scarce [35]. Moreover, their ATB-potentiating effect against *Staphylococcus* spp. biofilms has not yet been investigated. Therefore, the present study aimed to investigate the antibiofilm synergy and cytotoxicity of combining bioactive plant NPs with ATBs against three uropathogenic *Staphylococcus* species: *S. aureus*, *S. saprophyticus*, and *S. epidermidis*. Specifically, we sought to evaluate the individual and combined effects of selected bioactive plant NPs and ATBs on biofilm formation and eradication and assess the cytotoxicity of the best synergistic combinations on normal cell lines to determine their safety profile.

## 2. Materials and Methods

### 2.1. Chemicals

The ATBs used in this study included an aminopenicillin (amoxicillin), two cephalosporins (cefazolin and cefixime), two aminoglycosides (kanamycin and amikacin), a macrolide (erythromycin), and a tetracycline (doxycycline). The bioactive NPs used included curcumin, thymol, plumbagin, berberine, gallic acid, and quercetin. ATBs, natural compounds, and other chemicals such as 3-(4,5-dimethylthiazol-2-yl)-2,5-diphenyltetrazolium bromide (MTT), dimethyl sulfoxide (DMSO), and *p*-iodonitrotetrazolium chloride (INT) were purchased from Sigma-Aldrich (Germany).

### 2.2. Microorganisms and Cultures

Seven clinical isolates of *Staphylococcus* spp. including 2 *S. aureus*, 3 *S. saprophyticus*, and 2 *S. epidermidis* were used. They were isolated from urine samples in our previous study [36]. Two reference strains (*S. aureus* ATCC1026 and *S. epidermidis* ATCC35984) obtained from the American Type Culture Collection were also included. They were kept in the mixture of glycerol and Mueller Hinton Broth (MHB) (1:1) at −20°C, and the activation was achieved using the streak technique on Mueller Hinton agar (MHA) medium.

### 2.3. Determination of the Minimum Inhibitory Concentration (MIC) and the Minimum Bactericidal Concentration (MBC)

The MICs of ATBs and NPs were determined by the broth microdilution method as previously described [37]. NPs and ATBs were, respectively, prepared at 4096 µg/mL and 512 µg/mL and serially diluted twofold with MHB in a 96-well microplate at a volume of 100 µL. Then, the wells were filled with 100 µL of inoculum (1.5 × 10^6^ CFU/mL) to give final concentrations ranging from 1024 µg/mL to 0.5 µg/mL and 128 µg/mL to 0.062 µg/mL of NPs and ATBs, respectively. The microplates were incubated for 24 h at 37°C. Wells containing only bacterial inoculum were used as the negative control. After incubation, a volume of 40 µL of INT solution (0.2 mg/mL) was added to each well, and the microplate was incubated at 37°C for 30 min. Viable bacteria cells change the yellow dye of INT to a pink color. The MIC was considered as the lowest concentration of the sample at which there was no change in the coloration of the medium, corresponding to the complete inhibition of microbial growth.

The MBC was determined by adding 50 μL aliquots of the clear wells to 150 μL of freshly prepared MHB and incubating at 35°C for 48 h. The MBC was regarded as the lowest concentration of the test sample that did not produce a turbidity change as above. All tests were performed in triplicate.

### 2.4. Biofilm Inhibition Assay

The biofilm formation inhibitory activity of NPs and ATBs was carried out according to the method previously described [37]. A total of 100 µL of bacterial inoculum (1.5 × 10^6^ CFU/mL) was used, and 100 µL of each sample was added to the microplate to achieve final concentrations ranging from 0.25 µg/mL to 128 µg/mL for ATBs and from 8 µg/mL to 1024 µg/mL for NPs. Then, the microplate was incubated at 37°C for 24 h. After incubation, the plates were gently emptied and washed three times with a phosphate buffer solution at pH 7.2. Then, the MTT solution (0.5 mg/mL) prepared in PBS was introduced into each well, and the microplate was further incubated at 37°C for 24 h. Untreated wells containing bacterial suspension and MHB supplemented with 1% glucose were considered positive controls, and wells containing only MHB+1% glucose without bacteria were used as blanks. After incubation, the MTT solution was aspirated, then 150 µL of DMSO was introduced to dissolve the formazan crystal formed by viable bacteria, and the optical density (OD) was measured at 570 nm using a microplate reader (VERSA-max). The percentage of biofilm inhibition was calculated using the formula below, and the minimal biofilm inhibitory concentration (MBIC) was recorded as the lowest concentration of NPs or ATBs that inhibited 100% of biofilm:

(1) $$\begin{array}{c} \%\;b\text{iofilm inhibition}=\frac{\left({\text{OD}}_{\text{control}}-{\text{OD}}_{\text{blank}}\right)-\left({\text{OD}}_{\text{test}}-{\text{OD}}_{\text{blank}}\right)}{{\text{OD}}_{\text{control}-{\text{OD}}_{\text{blank}}}}\times 100. \end{array}$$

### 2.5. Biofilm Eradication Assay

The determination of the biofilm eradication potential of NPs and ATBs was performed as previously described [37]. Briefly, 100 µL of bacterial inoculum (1.5 × 10^6^ CFU/mL) and 100 µL of MHB + 1% glucose were introduced into the microplate, incubated at 37°C for 24 h, and the microplate was gently emptied and washed three times with PBS. Then, 200 µL of ATBs or NPs at respective concentrations ranging from 8 to 1024 µg/mL and from 16 to 2048 µg/mL were incubated at 37°C for 24 h. Once incubation was completed, the microplate was processed as described above, and the percentage of biofilm eradication was calculated using the formula below. The minimal biofilm eradicating concentration (MBEC) was recorded as the lowest concentration of the test sample that reduces 100% of biofilm:

(2) $$\begin{array}{c} \%\text{ biofilm eradication}=\frac{\left({\text{OD}}_{\text{control}}-{\text{OD}}_{\text{blank}}\right)-\left({\text{OD}}_{\text{test}}-{\text{OD}}_{\text{blank}}\right)}{{\text{OD}}_{\text{control}-{\text{OD}}_{\text{blank}}}}\times 100. \end{array}$$

### 2.6. Combination Studies Against Planktonic Cells of *Staphylococcus* spp.

The checkerboard assay, as previously described [37], was used for the determination of the fractional inhibitory concentration index (FICI). Briefly, 50 µL of MHB was distributed into each well of a 96-well microplate. ATBs were serially diluted along the abscissa, and NPs were serially diluted along the ordinate. Then, 100 µL of bacterial inoculum (1.5 × 10^6^ CFU/mL) was added to each well and incubated at 37°C for 24 h. The final concentration ranged from 0.125 to 128 µg/mL for ATBs and 4–512 µg/mL for NPs. After incubation, a volume of 40 µL of INT solution (0.2 mg/mL) was added to each well, and the microplate was incubated at 37°C for 30 min. The wells containing bacterial inoculum and MHB were used as a positive control, while wells containing only MHB + 1% glucose were used as a negative control and used as a blank. Viable bacteria cells change the yellow dye of INT to a pink color. The MICs were considered to be the lowest NP concentrations that prevented the color change medium. Subsequently, the FICI was calculated according to the formula FICI = (MIC of the ATBs in combination/MIC of the ATBs alone) + (MIC of a natural substance in combination/MIC of the natural substance alone). FICI values were interpreted as follows: synergy when FICI ≤ 0.5; additive when 0.5 < FICI ≤ 1; indifference when 1 < FICI ≤ 4; and antagonism when FICI > 4.

### 2.7. Combination Studies Against Biofilm Inhibition

The effect of the combination of ATBs with NPs on biofilm formation was evaluated by determining the FICI, as described above for planktonic cells, except that the MHB was supplemented with 1% glucose.

The lowest MBICs of ATBs or NPs that inhibited metabolic activity in the biofilm were determined as described above. The FICI was used to perform the effect of combination and calculated as follows: FICI: (MBIC of ATBs in combinations/MBIC of ATBs alone) + (MBIC of NP in the combinations/MBIC of NP alone). FICI was interpreted as indicated above.

### 2.8. Combination Studies Against Biofilm Eradication

The effect of the combination of ATBs with NPs on the preformed biofilm was evaluated by determining the FICI, as described above for biofilm formation, with slight modifications. After biofilm formation for 24 h, the plate was gently emptied and washed three times with PBS. Then, 50 µL of ATBs and 50 µL of NPs were introduced to the plate. The final concentration ranges from 0.0625 to 128 µg/mL and from 4 to 512 µg/mL for ATBs and NPs, respectively, and is incubated at 37°C for 24 h. After incubation, the medium was gently removed, and the plate was washed three times with PBS. At the end of the incubation time, the MTT reduction assay was performed, and the MBEC was determined. To assess the effect of the combination, the FICI was calculated and interpreted as described above.

### 2.9. Time-Kill Kinetic Assay on Biofilm Inhibition and Eradication

The five best synergistic antibiofilm combinations were selected, and their ability to inhibit biofilm formation and eradication as a function of time was evaluated at 4-h intervals for 24 h by the microtiter plate method, as previously described [26]. The synergistic antibiofilm combinations selected were with FICI < 0.4, named C1 to C5. C1: cefixime (5.33 µg/mL) + thymol (32 µg/mL); C2: cefazolin (1.16 µg/mL) + thymol (21.33 µg/mL); C3: amikacin (0.18 µg/mL) + curcumin (37.33 µg/mL); C4: kanamycin (0.25 µg/mL) + curcumin (14 µg/mL); and C5: amoxicillin (1.16 µg/mL) + curcumin (21.33 µg/mL). The kinetics of biofilm inhibition and eradication were performed as described above for the biofilm inhibition and eradication assay using the indicated combinations, followed by the reading of the optical densities (570 nm) at 4-h intervals over 24 h (0, 4, 8, 12, 16, and 24 h). In both cases, experiments were carried out three times in triplicate.

### 2.10. Cytotoxic Activity

#### 2.10.1. Cell Lines and Culture Conditions

Two cell lines, including Raw 264.7 macrophages and the urinary epithelial cell line UROtsa obtained from the American Type Culture Collection (Rockville, MD, USA), were used. They were cultured in Dulbecco's modified Eagle's medium (DMEM), which was supplemented with 10% fetal bovine serum (FBS) and 1% penicillin, streptomycin, and fungizone (PSF). They were then grown at 37°C in a humidified environment containing 5% CO_2_.

#### 2.10.2. MTT Assay

The cytotoxicity of the selected synergistic combinations, as well as the individual constituents of combinations, against the abovementioned cell lines was performed by the MTT assay as previously described [38]. Briefly, cells from each line were harvested in the logarithmic phase using trypsin (0.05% trypsin, 0.02% EDTA in PBS). Cell suspension was diluted with an appropriate growth medium to obtain a cell density of 10^4^ cells/well. A 100 µL aliquot of each suspension was seeded into 96-well cell culture plates and incubated at 37°C in an atmosphere of 5% CO_2_ and 95% relative humidity. After 24 h of incubation, 100 µL of samples (ATBs and NPs) at concentrations varying from 0.01, 0.1, 1, 10, 100, and 1000, as well as the synergistic combinations (C1 to C5), was added to the wells containing the cells. Doxorubicin was used as a positive control. Appropriate controls with an equivalent concentration of DMSO were also included. Plates were incubated for 48 h, then the medium in each well was aspirated, and MTT solution (2 mg/mL in PBS) was diluted 1:10 with fresh medium and added to each well, and the plates were further incubated at 37°C for 4 h. Absorbance was measured on a BioTek microplate reader at 570 nm. The percentage of cell viability was calculated by comparing treated and untreated cells using the equation [39]:

(3) $$\begin{array}{c} \text{cell viability }\left(\%\right)=\left[\frac{{\text{OD}}_{\text{test}}}{{\text{OD}}_{\text{control}}}\right]\times 100. \end{array}$$

Then, the IC_50_ values of the samples having shown at least 50% inhibition were calculated by plotting the percentage of inhibition against the concentrations.

#### 2.10.3. Data Analysis

The synergistic effect was analyzed using Microsoft Excel 2016. In addition, GraphPad Prism 8.0.1 software was used to analyze the difference between the activities of the test products and those of the untreated control, as well as to analyze cytotoxic activities with the one-way and two-way ANOVA tests. Differences were considered significant at *p* < 0.05.

## 3. Results

### 3.1. The Anti-Staphylococcal Activity of ATBs and NPs

The MICs and MBCs of NPs and ATBs against strains of *Staphylococcus* spp. were evaluated, and the results are presented in Table 1. Among NPs, plumbagin exhibited strong anti-staphylococcal properties, particularly against *S. aureus* (MIC: 6 µg/mL) and *S. saprophyticus* (MIC: 8.66 µg/mL), while gallic acid showed significantly weaker activity (MIC: 426.66 µg/mL against the three *Staphylococcus* species). ATBs generally exhibited lower MIC values, indicating good antibacterial activity, with amikacin (MIC: 1.16 µg/mL and 4.33 µg/mL against *S. epidermidis* and *S. aureus,* respectively), erythromycin (MIC: 4.66 µg/mL against *S. saprophyticus*), and doxycycline (MIC: 6.16 µg/mL against *S. aureus*) demonstrating strong efficacy.

### 3.2. Antibiofilm Activity of NPs and ATBs

Table 2 presents the MBICs and MBECs values of NPs and ATBs against the three *Staphylococcus* species tested. The results demonstrated that ATBs generally exhibited better antibiofilm activity compared to NPs, indicating higher efficacy in inhibiting and eradicating biofilms of *Staphylococcus* spp. Among NPs, plumbagin showed the most potent activity, with MBIC values as low as 6.66 µg/mL against *S. saprophyticus* and MBEC values as low as 10.66 µg/mL against *S. epidermidis*. In contrast, other compounds such as gallic acid and quercetin exhibited significantly higher MBIC and MBEC values, suggesting weaker biofilm inhibition and eradication potential. Thymol and curcumin also demonstrated moderate biofilm inhibition and eradication properties. Among ATBs, amikacin, amoxicillin, and doxycycline exhibited the lowest MBIC and MBEC values, confirming their strong antibiofilm effects. Notably, cefixime showed relatively higher MBIC and MBEC values, indicating a weaker ability to inhibit and eradicate biofilms.

### 3.3. Effect of Combining NPs and ATBs Against Planktonic Cells of *Staphylococcus* spp.

The result of the synergistic interactions obtained from the combination of NPs with ATBs against planktonic cells of *Staphylococcus* spp. is shown in Table 3. It appears that the MIC values of ATBs significantly decreased when combined with NPs, confirming their potential as ATB enhancers. The MIC reduction fold for ATBs ranged from 2.94 to 7. The FICI values (≤ 0.5) confirm synergy in all tested combinations. Other types of interaction (additivity and indifference) effects were also obtained from the combinations, and results are presented as Supporting Information (S1–S3).

### 3.4. Effect of Combining NPs and ATBs Against the Inhibition of *Staphylococcus* Species Biofilm

Data in Table 4 reveal the results of synergistic interactions between ATBs and NPs in inhibiting biofilm formation by *Staphylococcus* spp. The results indicate a substantial reduction in MBIC values when ATBs are combined with NPs, highlighting their potential as biofilm-targeting agents. The observed MBIC reduction fold ranges from 2.28 to 18.18. The combination of amikacin with curcumin against *S. epidermidis* demonstrated a 16.44-fold reduction in MBIC, reinforcing the efficacy of curcumin as a biofilm inhibitor. Similarly, the combination of kanamycin with curcumin against *S. saprophyticus* resulted in a 15.07-fold reduction. The results of the other types of interaction obtained are presented as Supporting Information (S4–S6).

### 3.5. Effect of Combining NPs and ATBs Against the Eradication of *Staphylococcus* Species Biofilm

The synergistic effects of combining NPs with ATBs against biofilm eradication are shown in Table 5. All the combinations presented demonstrated a synergistic effect, as indicated by FICI values below 0.5. The most significant MBEC reduction was observed in the combination of cefazolin with thymol against *S. saprophyticus*, where the MBEC of cefazolin decreases from 48 µg/mL to 5.33 µg/mL, with a remarkable reduction fold of 9. Plumbagin, in combination with cefixime and doxycycline, effectively reduced the MBEC values of both ATBs against *S. saprophyticus* and *S. epidermidis*, respectively. Other types of interaction (additivity and indifference) effects were also obtained from the combinations, and results are presented as Supporting Information (S7–S9).

### 3.6. Time-Kill Kinetic Study of Selected Synergistic Combinations

#### 3.6.1. Time-Kill Kinetic Study of Selected Combinations on Biofilm Inhibition

**Figure 1:**
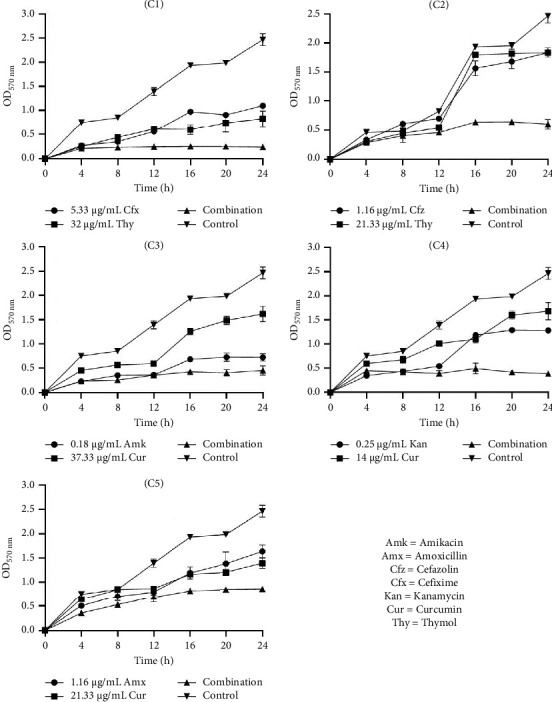
Time-kill kinetic study of selected antibiotics and natural products alone and in combination against the inhibition of *S. aureus* biofilm. C1: cefixime (5.33 μg/mL) + thymol (32 μg/mL), C2: cefazolin (1.16 μg/mL) + thymol (21.33 μg/mL), C3: amikacin (0.18 μg/mL) + curcumin (37.33 μg/mL), C4: kanamycin (0.25 μg/mL) + curcumin (14 μg/mL), and C5: amoxicillin (1.16 μg/mL) + curcumin (21.33 μg/mL).

**Figure 2:**
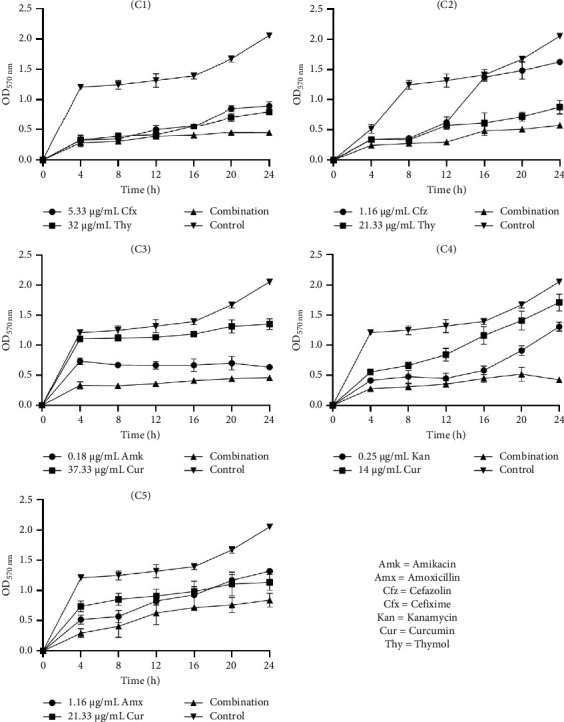
Time-kill kinetic study of selected antibiotics and natural products alone and in combination against the inhibition of *S. saprophyticus* biofilm. C1: cefixime (5.33 μg/mL) + thymol (32 μg/mL), C2: cefazolin (1.16 μg/mL) + thymol (21.33 μg/mL), C3: amikacin (0.18 μg/mL) + curcumin (37.33 μg/mL), C4: kanamycin (0.25 μg/mL) + curcumin (14 μg/mL), and C5: amoxicillin (1.16 μg/mL) + curcumin (21.33 μg/mL).

**Figure 3:**
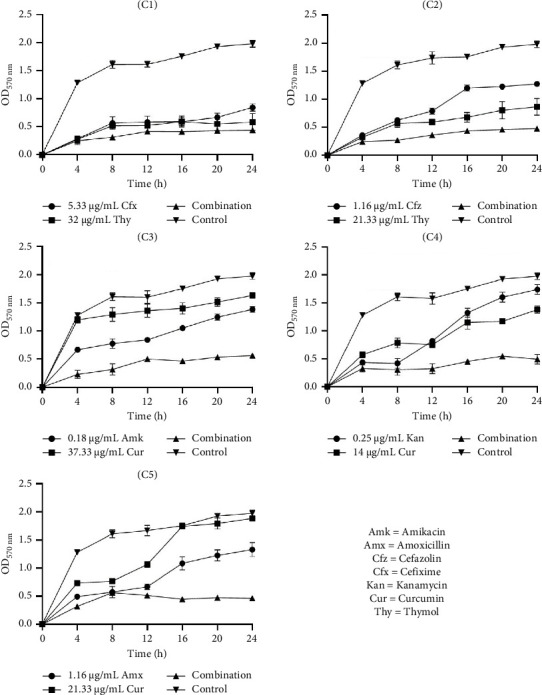
Time-kill kinetic study of selected antibiotics and natural products alone and in combination against the inhibition of *S. epidermidis* biofilm. C1: cefixime (5.33 μg/mL) + thymol (32 μg/mL), C2: cefazolin (1.16 μg/mL) + thymol (21.33 μg/mL), C3: amikacin (0.18 μg/mL) + curcumin (37.33 μg/mL), C4: kanamycin (0.25 μg/mL) + curcumin (14 μg/mL), and C5: amoxicillin (1.16 μg/mL) + curcumin (21.33 μg/mL).

The data in Figures 1, 2, and 3 present the efficacy of ATB and NP combinations in inhibiting *S. aureus*, *S. saprophyticus,* and *S. epidermidis* biofilms over 24 h. In all cases, control biofilms exhibited substantial growth (OD up to 2.46 for *S. aureus* and 1.98 for *S. epidermidis*), while ATB-NP combinations significantly suppressed OD, highlighting their enhanced efficacy compared to antimicrobials alone.

For *S. aureus* (Figure 1), thymol paired with β-lactams (cefixime or cefazolin) showed remarkable synergy, reducing OD to 0.25–0.60 at 24 h versus 1.10–1.84 for individual antimicrobials. Curcumin combined with aminoglycosides (amikacin/kanamycin) also reduced biofilm formation. In *S. saprophyticus* (Figure 2), all ATB-NP combinations showed lower OD values compared to antimicrobials alone. Thymol-based combinations (C1 and C2) reduced OD to 0.45–0.58 and to 0.84–1.62 for single agents. Curcumin-based combinations (C3, C4, and C5) synergized strongly with aminoglycosides. Against *S. epidermidis* (Figure 3), combination C1 (cefixime + thymol) exhibited moderate biofilm inhibition, with OD values reaching 0.44 at 24 h compared to 0.84 (ATB alone) and 0.67 (NP alone). Combination C2 (cefazolin + thymol) demonstrated superior efficacy, reducing OD to 0.48 at 24 h compared to 1.27 (ATB alone) and 0.96 (NP alone). Curcumin-containing combinations (C3, C4, and C5) had significant effects.

#### 3.6.2. Time-Kill Kinetic Study of Selected Combinations on Biofilm Eradication

**Figure 4:**
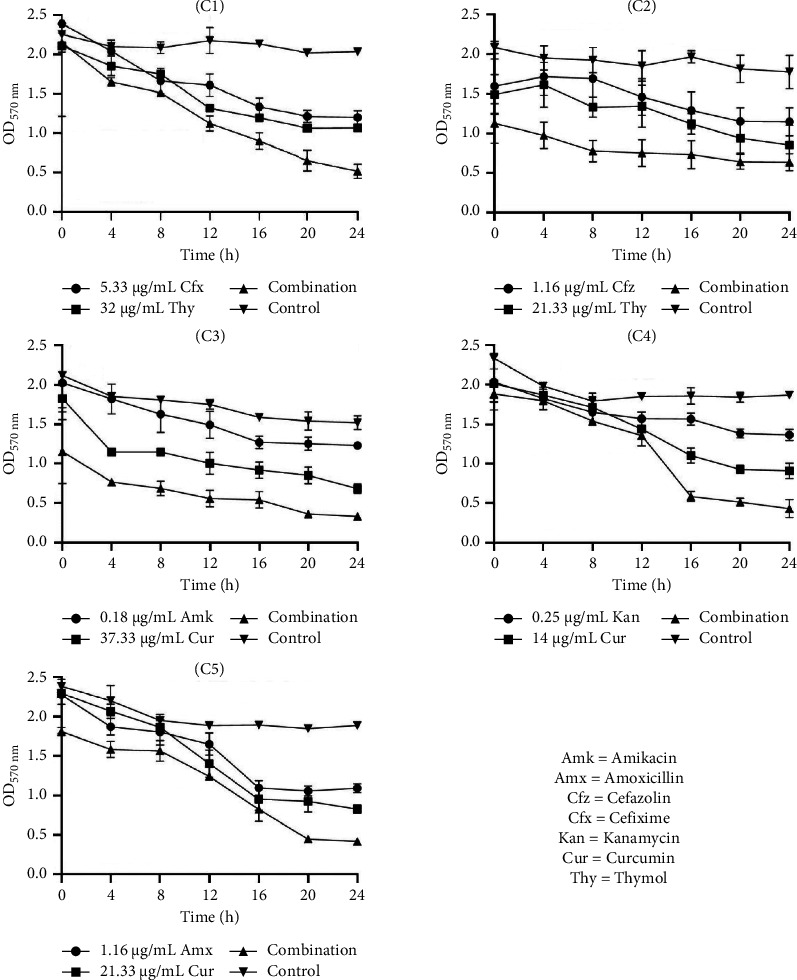
Time-kill kinetic study of selected antibiotics and natural products alone and in combination against the eradication of *S. aureus* biofilm. C1: cefixime (5.33 μg/mL) + thymol (32 μg/mL), C2: cefazolin (1.16 μg/mL) + thymol (21.33 μg/mL), C3: amikacin (0.18 μg/mL) + curcumin (37.33 μg/mL), C4: kanamycin (0.25 μg/mL) + curcumin (14 μg/mL), and C5: amoxicillin (1.16 μg/mL) + curcumin (21.33 μg/mL).

**Figure 5:**
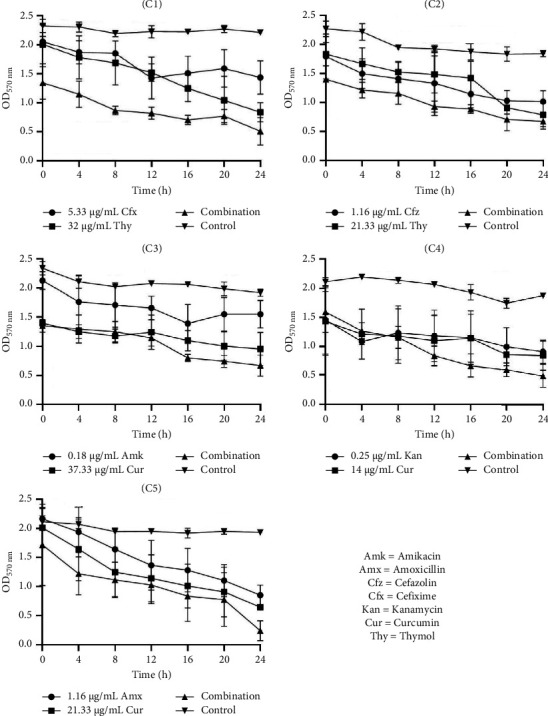
Time-kill kinetic study of selected antibiotics and natural products alone and in combination against the eradication of *S. saprophyticus* biofilm. C1: cefixime (5.33 μg/mL) + thymol (32 μg/mL), C2: cefazolin (1.16 μg/mL) + thymol (21.33 μg/mL), C3: amikacin (0.18 μg/mL) + curcumin (37.33 μg/mL), C4: kanamycin (0.25 μg/mL) + curcumin (14 μg/mL), and C5: amoxicillin (1.16 μg/mL) + curcumin (21.33 μg/mL).

**Figure 6:**
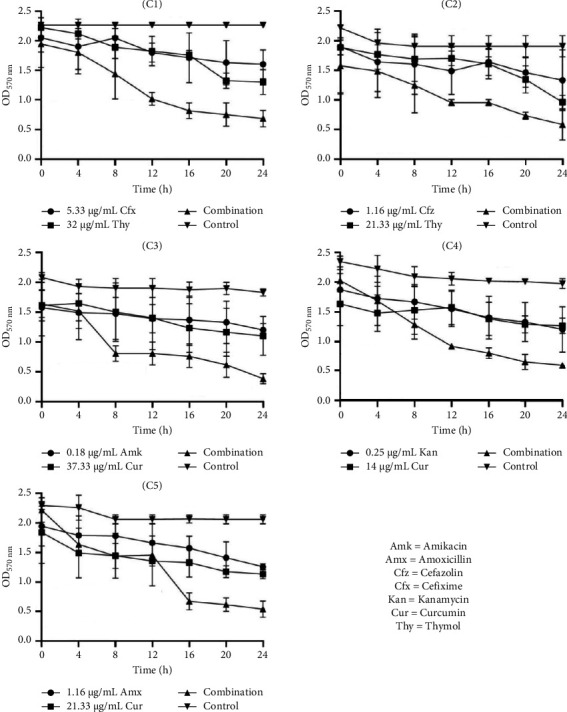
Time-kill kinetic study of selected antibiotics and natural products alone and in combination against the eradication of *S. epidermidis* biofilm. C1: cefixime (5.33 μg/mL) + thymol (32 μg/mL), C2: cefazolin (1.16 μg/mL) + thymol (21.33 μg/mL), C3: amikacin (0.18 μg/mL) + curcumin (37.33 μg/mL), C4: kanamycin (0.25 μg/mL) + curcumin (14 μg/mL), and C5: amoxicillin (1.16 μg/mL) + curcumin (21.33 μg/mL).

Data presented in Figures 4, 5, and 6 highlight the enhanced efficacy of combining ATBs with NPs in eradicating biofilms of *S. aureus*, *S. saprophyticus*, and *S. epidermidis*. OD measurements indicate bacterial biofilm presence, with lower values signifying greater eradication. In general, the selected combination of NPs with ATBs consistently exhibited higher antibiofilm eradication compared to individual ATB or NP treatment.

In *S. aureus* studies (Figure 4), combination C1 (cefixime [5.33 µg/mL] + thymol [32 µg/mL]) showed a marked OD reduction from 2.15 to 0.52 over 24 h, while combination C3 (amikacin [0.18 µg/mL] + curcumin [37.33 µg/mL]) achieved the most significant drop to 0.33. For *S. epidermidis* (Figure 6), the five combinations effectively reduced biofilm, with the most effective eradication occurring at 24 h, showing optical densities around 0.5. and 1.10).

### 3.7. Comparative Analyses of Differences Between the Antibiofilm Activity (Inhibitors/Eradicators) of Synergistic Combinations and Their Individual Components Compared With the Untreated Control

The one-way ANOVA test was used to compare the antibiofilm (inhibition/eradication) activities of the five combinations (C1, C2, C3, C4, and C5) on *S. aureus*, *S. saprophyticus,* and *S. epidermidis* strains, respectively, and the result is presented in Table 6. Compared to the untreated control, each combination showed a significant difference in antibiofilm activities against each of the three *Staphylococcus* species. Eradicating activities were more significant than inhibitory activities. The antibiofilm activities of the two compounds (curcumin and thymol) of each combination taken separately showed less significant differences than those of the combination.

### 3.8. Cytotoxic Effect of Selected ATBs and NPs, Alone and in Combination on UROtsa and Raw 264.7 Cell Lines

Table 7 presents the IC_50_ values (concentration required to inhibit 50% of cell viability) of the investigated ATBs and NPs on two cell lines: UROtsa (a noncancerous urinary epithelial cell line) and Raw 264.7 (a macrophage cell line). The ATBs tested (amoxicillin, amikacin, cefixime, kanamycin, and cefazolin) showed no significant cytotoxicity on both cell lines, as indicated by IC_50_ values greater than 1000 µg/mL, suggesting they are relatively safe at the concentrations tested. The NPs, curcumin and thymol, showed moderate cytotoxicity, with IC_50_ values ranging from 51.89 µg/mL (UROtsa) to 94.40 µg/mL (Raw 264.7) for curcumin and 441.78 µg/mL (Raw 264.7) for thymol, indicating that these compounds are less toxic than doxorubicin. Doxorubicin, a chemotherapeutic agent, is highly cytotoxic to both cell lines with IC_50_ values of 1.58 µg/mL (UROtsa) and 1.26 µg/mL (Raw 264.7), emphasizing its potent cell-killing ability.

### 3.9. Cytotoxicity of the Selected Synergistic Combinations on UROtsa and Raw 264.7 Cell Lines

**Figure 7:**
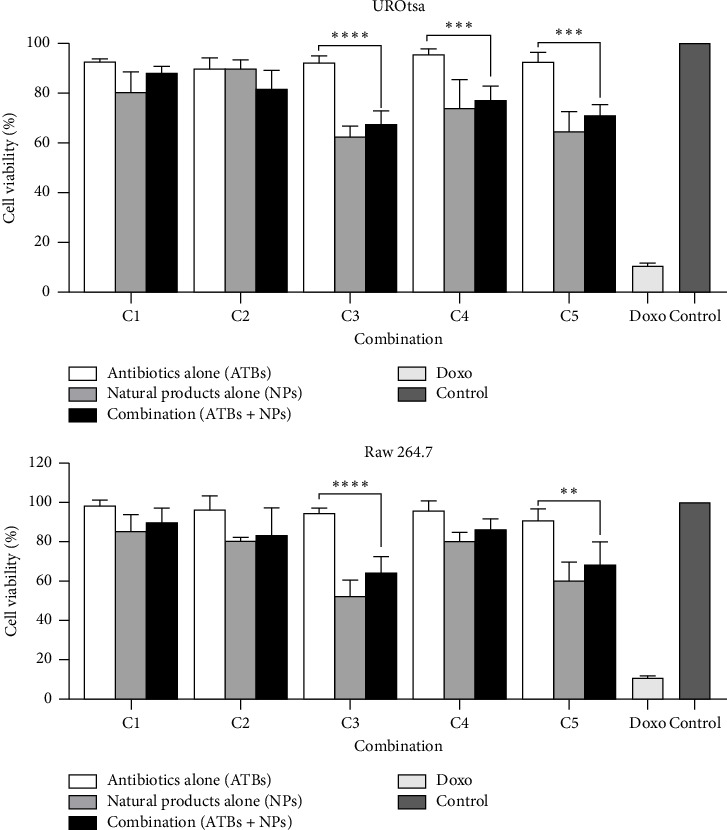
Effect of synergistic combinations on UROtsa cell viability. C1: cefixime (5.33 μg/mL) + thymol (32 μg/mL), C2: cefazolin (1.16 μg/mL) + thymol (21.33 μg/mL), C3: amikacin (0.18 μg/mL) + curcumin (37.33 μg/mL), C4: kanamycin (0.25 μg/mL) + curcumin (14 μg/mL), and C5: amoxicillin (1.16 μg/mL) + curcumin (21.33 μg/mL). Doxo: doxorubicin (2.5 μg/mL). Statistical analyses were performed with Dunnett's multiple comparison test using two-way ANOVA; ^∗^*p* < 0.01; ^∗∗^*p* < 0.001; ^∗∗∗^*p* < 0.001; ^∗∗∗∗^*p* < 0.0001.

Figure 7 presents the effects of various combinations of ATBs and NPs on RAW 264.7 cell viability. The results indicate that ATB generally maintains higher RAW 264.7 cell viability compared to NPs alone or their combination (ATB + NPs). Across combinations C1 to C5, cell viability with ATB ranged from 90.05% to 95.66% for UROtsa and from 90.71% to 98.18% for Raw 264.7 cells, whereas NPs alone or in combination with ATB (ATB + NPs) showed lower viability percentages ranging from 62.57% to 89.85% for UROtsa and from 52.30% to 85.38% for Raw 264.7 cells. Notably, C3 exhibits a significant drop in cell viability from 92.46% with ATB alone to 67.42% when combined with NPs. Similarly, C3 exhibits the largest disparity, with ATB alone at 94.43% versus a significantly lower 64.58% when combined with NPs, suggesting a potential antagonistic effect of NPs on cell health. These results suggest that while the combinations may have some cytotoxic effects, their impact on UROtsa cells remains within a moderate range, implying potential selective effects on immune cells versus epithelial cells.

## 4. Discussion

*Staphylococcus* species, particularly *S. saprophyticus*, *S. epidermidis*, and *S. aureus,* are significant uropathogens responsible for UTIs. Their ability to form biofilms on urinary catheters, bladder surfaces, and medical implants makes infections persistent and difficult to eradicate [40]. To address this challenge, combination therapy involving conventional ATBs and adjunctive agents such as NPs, nanoparticles, and quorum-sensing inhibitors is emerging as a promising strategy. In this study, we assessed the synergistic effects of combining bioactive NPs (curcumin, thymol, plumbagin, berberine, gallic acid, and quercetin) with conventional ATBs (amoxicillin, cefazolin, cefixime, kanamycin, amikacin, erythromycin, and doxycycline) against *Staphylococcus* spp. planktonic cells and biofilm. In addition, the cytotoxicity of the best synergistic combinations was also evaluated against urothelial UROtsa and Raw 264.7 macrophage cell lines.

The MIC and MBC values obtained indicated varying levels of antimicrobial activity among NPs and ATBs against *Staphylococcus* spp.; particularly, plumbagin exhibited low MIC values against *S. aureus* and *S. saprophyticus* as previously reported [41]. The MIC values for ATBs were generally lower, with amikacin, doxycycline, and kanamycin, demonstrating their role as standard treatments for *Staphylococcus* infections [42] and further suggesting their potential effectiveness in combating biofilm-forming strains, as biofilms are notoriously recalcitrant to conventional ATBs [43].

The ability of NPs and ATBs to prevent biofilm formation and to disrupt the preformed biofilm of *Staphylococcus* species was also evaluated. The data suggest that while NPs displayed some biofilm inhibitory and eradication capabilities, their effectiveness was generally lower than that of conventional ATBs [44]. ATBs such as amikacin and doxycycline demonstrate the lowest MBIC and MBEC values across all tested *Staphylococcus* species, reinforcing their role as effective biofilm-targeting agents [45]. These ATBs are known to penetrate biofilm structures effectively and exert bactericidal effects against dormant bacterial cells [46]. The strong activity of plumbagin suggests its potential as an antibiofilm agent. Recent studies highlighted the ability of plumbagin to disrupt bacterial biofilm [47]. The high MBIC and MBEC values of certain NPs indicate that they may require higher concentrations or combination therapies to achieve significant biofilm reduction. These findings recommend that while NPs alone may not be sufficient for bacterial growth inhibition, or bacterial biofilm eradication, they could serve as adjunct therapies in combination with ATBs to enhance antimicrobial efficacy and combat resistance. Therefore, in this study, we further explore synergistic approaches between NPs and ATBs against planktonic cells of *Staphylococcus* species, as well as to prevent biofilm formation and enhance biofilm eradication efficacy.

The observed MIC reduction suggests that NPs may increase bacterial susceptibility to ATBs, possibly by disrupting bacterial cell structures or resistance mechanisms, such as efflux pump inhibition or membrane permeability alteration [48]. Doxycycline-based combinations, particularly with thymol and gallic acid, demonstrated remarkable efficacy, suggesting these compounds could improve ATB potency by targeting bacterial protein synthesis pathways while destabilizing biofilms [49]. The ability of curcumin, thymol, and gallic acid to lower MIC values aligns with studies identifying their role as effective adjuvants through mechanisms like reactive oxygen species generation and quorum sensing interference [50]. The synergistic effects observed may also stem from the multitarget mechanisms of plant NPs. For instance, curcumin, known for its antioxidant properties, likely enhances ATB efficacy by mitigating bacterial oxidative stress responses [51]. Thymol disrupts membrane integrity, while berberine inhibits efflux pumps, collectively lowering the effective ATB dose required for biofilm eradi. Other work has shown the synergistic effects of combining kanamycin with thymol against biofilm-associated *Salmonella enterica* [26]. Also, the combination of curcumin and gentamicin showed synergistic antibacterial effects [52]. Curcumin has the potential to reduce biofilm initiation genes, inhibit quorum sensing genes, and downregulate virulence factors, including elastase/protease production and pyocyanin synthesis [53]. Furthermore, curcumin has been reported to inhibit biofilm formation by targeting regulatory genes involved in bacterial adhesion, while thymol disrupts membrane integrity, increasing bacterial susceptibility to ATBs [54]. Additionally, the reduction in MBIC values suggests a significant improvement in biofilm susceptibility, which is critical in clinical settings where persistent infections pose therapeutic challenges [45]. Recent studies highlight that natural compounds can disrupt biofilm structure, interfere with quorum sensing, and enhance ATB penetration, making them valuable adjuvants in antimicrobial treatments [55].

The significant reduction in MBEC values observed in this study suggests that NPs may enhance bacterial susceptibility, likely by disrupting biofilm structure or interfering with resistance mechanisms [44]. Notably, combinations such as cefazolin + thymol and erythromycin + berberine demonstrated strong efficacy, making them promising candidates for further exploration. Thymol has been reported to destabilize bacterial membranes and inhibit quorum sensing, while berberine interferes with efflux pumps and cell signaling pathways, enhancing ATB action [56]. Thymol is classified by the FDA as generally safe [57]. These findings align with recent studies highlighting the ability of phytochemicals to potentiate ATBs against biofilm-forming pathogens [58]. NPs can exert their synergistic action through several strategies, such as the inhibition of target-modifying and drug-degrading enzymes such as efflux pumps or by facilitating their entry into the cell by altering the cytoplasmic membrane or dispersing biofilms [59].

As biofilm formation is crucial for staphylococcal infections, synergistic antibiofilm activity over time is an important feature linked to potential therapeutic application in the control of staphylococcal infections [60]. The combinations showed suboptimal biofilm inhibitory and eradicating activities from 16 h to 24 h, with optical densities below 0.5. Previous studies have reported the kinetics of thymol in combination with streptomycin and gentamicin showed total growth inhibition between 0- and 24-h incubation with optical densities below 0.2 [57].

Any antibacterial drug candidate is required to have a high benefit/risk ratio [61]. Therefore, cytotoxicity evaluation is considered a preliminary test to ensure product safety [62]. In this study, the cytotoxicity of selected synergistic combinations was also assessed on Raw 264.7 macrophage and UROtsa urinary epithelium cells. The observed trends in cell viability suggested that NPs contribute significantly to cytotoxicity, particularly in RAW 264.7 macrophages, which play a crucial role in immune defense [63]. The reduced viability in RAW 264.7 cells when treated with NPs raises concerns about potential immunosuppressive effects, as macrophages are critical for pathogen clearance and inflammatory regulation [64]. However, the combined treatment partially restored cell viability, proposing a possible protective or antagonistic interaction between the two combined agents. These findings indicated that while combinations involving ATB and NPs may have therapeutic potential, their safety profile should be thoroughly assessed, especially concerning immune system suppression and epithelial cell integrity.

## 5. Conclusion

The increasing prevalence of biofilm-related infections among uropathogenic *Staphylococcus* spp. highlights the urgent need for novel therapeutic approaches. This study explores the potential of bioactive plant NPs to enhance the efficacy of conventional ATBs, offering a dual benefit of overcoming biofilm-associated resistance and reducing the effective doses of ATBs required for treatment. Additionally, the findings of this research may contribute to the development of safer and more effective combination therapies, ultimately improving clinical outcomes for patients with UTI-related infections. Future research should focus on elucidating the mechanisms of action, optimizing these combinations for clinical application, and conducting *in vivo* studies to validate the results.

## Figures and Tables

**Table 1:** MIC and MBC geometric means of natural products and antibiotics against three species of *Staphylococcus*.

Antimicrobial agents	MICs/MBCs (µg/mL)		
	*S. aureus* (*n* = 3)	*S. saprophyticus* (*n* = 3)	*S. epidermidis* (*n* = 3)
Curcumin	243.66/—	170.66/—	384/—
Berberine	138.66/—	149.33/—	213.33/—
Gallic acid	426.66/—	426.66/—	426.66/—
Plumbagin	6/—	8.66/—	90.66/—
Quercetin	341.33/—	341.33/—	512/—
Thymol	298.66/—	341.33/—	341.33/—
Amikacin	4.33/—	22/—	1.16/—
Amoxicillin	86.66/—	22/—	26.83/—
Cefazolin	42.66/—	64.66/—	42.66/138.66
Cefixime	213.33/—	192/—	106.66/—
Doxycycline	6.16/32.66	32.16/—	7/8
Erythromycin	108/—	4.66/—	32.66/—
Kanamycin	22.66/69.33	14/—	7.33/12

*Note:* —: MIC or MBC > 1024 µg/mL for natural product and > 128 µg/mL for ATBs.

**Table 2:** MBIC and MBEC geometric means of natural products and antibiotics against three species of *Staphylococcus*.

Antimicrobial agents	MBICs and MBECs (µg/mL)					
	*S. aureus* (*n* = 3)		*S. saprophyticus* (*n* = 3)		*S. epidermidis* (*n* = 3)	
	MBIC	MBEC	MBIC	MBEC	MBIC	MBEC
Curcumin	106.66	149.33	106.66	170.66	128	106.66
Berberine	170.66	149.33	85.33	170.66	256	170.66
Gallic acid	512	298.66	426.66	298.66	512	266.66
Plumbagin	10.66	32	6.66	10.66	8	10.66
Quercetin	512	128	426.66	170.66	512	298.66
Thymol	341.33	106.66	128	106.66	213.33	106.66
Amikacin	1.83	13.33	2.08	13.33	3.08	21.33
Amoxicillin	1.5	37.33	9	9.33	2.08	16
Cefazolin	4.66	42.66	8.53	10.66	8.66	10.66
Cefixime	64	42.66	48	48	64	42.66
Doxycycline	2.16	16	0.5	9.33	3.08	16
Erythromycin	42.83	42.83	27.33	29.33	24	18.66
Kanamycin	1.08	14.66	4.08	10.66	24.66	13.33

**Table 3:** Mean of the minimum inhibitory concentration (MIC), fractional inhibitory concentration (FIC), and fractional inhibitory concentration index (FICI) of natural products (NPs) and antibiotics (ATB) in combination against *Staphylococcus* spp.

Antimicrobial agents in combination	MIC (µg/mL)				FIC		MIC reduction fold of ATB	FICI/interpretation
	Alone		Combined					
	ATB	NPs	ATB	NPs	ATB	NPs		
Amk + Cur	1.16	384	0.39	53.33	0.33	0.13	2.94	0.47/Syn
Dox + Thy	7	426.66	1	138.66	0.14	0.32	7	0.46/Syn
Dox + Gal	7	512	1.04	128	0.14	0.25	6.72	0.39/Syn

*Note:* Syn: synergism, Amk: amikacin, Dox: doxycycline, Cur: curcumin, Thy: thymol, Gal: gallic acid.

**Table 4:** Mean of the minimum biofilm inhibitory concentration (MBIC), fractional inhibitory concentration (FIC), and fractional inhibitory concentration index (FICI) of natural products (NPs) and antibiotics (ATBs) in combination against *Staphylococcus* spp.

Antimicrobial agents in combination	MBIC (µg/mL)				FIC		MBIC reduction fold	FICI/interpretation
	Alone		Combined					
	ATB	NPs	ATB	NPs	ATB	NPs		
*Staphylococcus aureus*								
Amk + Thy	1.83	341.33	0.45	34.66	0.25	0.10	4	0.35/Syn
Cfz + Plu	4.66	10.66	1.16	2.33	0.25	0.21	4	0.46/Syn
Kan + Thy	1.83	341.33	0.35	69.33	0.19	0.20	5.17	0.39/Syn

*Staphylococcus saprophyticus*								
Amk + Cur	2.08	106.66	0.52	13.33	0.25	0.12	4	0.37/Syn
Amk + Thy	2.08	128	0.37	29.33	018	0.22	5.55	0.40/Syn
Dox + Plu	0.5	16	0.21	8.33	0.43	0.52	2.28	0.43/Syn
Dox + Thy	0.5	128	0.19	13.33	0.39	0.10	2.52	0.5/Syn
Cfz + Cur	8.33	106.66	1.16	33.33	0.14	0.31	7.14	0.45/Syn
Cfz + Thy	8.33	128	1.16	21.33	0.14	0.16	7.14	0.30/Syn
Amx + Cur	8.33	106.66	1.16	21.33	0.14	0.2	7.14	0.34/Syn
Amx + Ber	8.33	106.66	0.45	37.33	0.05	0.35	18.18	0.40/Syn
Kan + Cur	4.08	106.66	0.27	14	0.06	0.13	15.07	0.19/Syn

*Staphylococcus epidermidis*								
Amk + Cur	3.08	128	0.18	37.33	0.06	0.29	16.44	0.35/Syn
Cfz + Cur	8.66	128	1.16	37.33	0.13	0.29	7.42	0.42/Syn
Cfz + Thy	8.66	213.33	0.83	74.66	0.09	0.35	10.4	0.44/Syn
Amx + Cur	2.08	128	0.16	42.66	0.08	0.33	12.5	0.41/Syn
Amx + Plu	2.08	8	0.20	3	0.1	0.37	10	0.47/Syn
Kan + Ber	24.66	256	8.16	64	0.33	0.25	3.02	0.5/Syn

*Note:* Syn: synergistic effect, Amk: amikacin, Amx: amoxicillin, Cfz: cefazolin, Cfx: cefixime, Kan: kanamycin, Gal: gallic acid, Ber: berberine, Cur: curcumin, Thy: thymol.

**Table 5:** Mean of the minimum biofilm eradication concentration (MBEC), fractional inhibitory concentration (FIC), and fractional inhibitory concentration index (FICI) of natural products (NPs) and antibiotics (ATBs) in combination against *Staphylococcus* spp.

Antimicrobial agents in combination	MBEC (µg/mL)				FIC		MBEC reduction fold	FICI/interpretation
	Alone		Combined					
	ATB	NPs	ATB	NPs	ATB	NPs		
*Staphylococcus aureus*								
Amk + Ber	13.33	149.33	3.33	32	0.25	0.21	4	0.46/Syn
Cfz + Cur	42.66	277.33	10.06	53.33	0.25	0.19	4	0.44/Syn

*Staphylococcus saprophyticus*								
Ery + Ber	29.33	128	4.66	42.66	0.15	0.33	6.28	0.49/Syn
Cfx + Plu	48	10.66	10.66	5.33	0.22	0.5	4.5	0.50/Syn
Cfz + Thy	48	128	5.33	32	0.066	0.25	9	0.31/Syn

*Staphylococcus epidermidis*								
Dox + Plu	16	13.33	4.66	2.66	0.29	0.2	3.43	0.49/Syn
Cfz + Plu	42.66	16	10.66	4	0.25	0.25	4	0.5/Syn

*Note:* Syn: synergistic effect, Amk: amikacin, Amx: amoxicillin, Cfz: cefazolin, Cfx: cefixime, Kan: kanamycin, Gal: gallic acid, Ber: berberine, Cur: curcumin, Thy: thymol.

**Table 6:** Summary of *p* values obtained from the ordinary one-way ANOVA test between the biofilm inhibition and eradication kinetics of each of the antimicrobial agents tested alone and in combination against *Staphylococcus* spp. compared with the untreated biofilm control.

Combinations		ANOVA one-way *p* value					
		*S. aureus*		*S. saprophyticus*		*S. epidermidis*	
		Inhibition	Eradication	Inhibition	Eradication	Inhibition	Eradication
C1	Cfx-control	0.0273^∗^	0.1140^ns^	0.0026^∗∗^	0.0024^∗∗^	0.0004^∗∗∗^	0.049^∗^
	Thy-control	0.0117^∗^	0.0269^∗^	0.0016^∗∗^	< 0.0001^∗∗∗∗^	0.0002^∗∗∗^	0.0286^∗^
	C1-control	0.0008^∗∗∗^	0.0017^∗∗^	0.0004^∗∗∗^	< 0.0001^∗∗∗∗^	< 0.0001^∗∗∗∗^	< 0.0001^∗∗∗∗^
C2	Cfz-control	0.9184^ns^	0.001^∗∗^	0.4559^ns^	0.007^∗∗∗^	0.0242^∗^	0.0368^∗^
	Thy-control	0.9224^ns^	< 0.0001^∗∗∗∗^	0.0503^ns^	0.0016^∗∗^	0.0024^∗∗^	0.0287^∗^
	C2-control	0.1773^ns^	< 0.0001^∗∗∗∗^	0.0143^∗^	< 0.0001^∗∗∗∗^	0.0033^∗∗∗^	< 0.0001^∗∗∗∗^
C3	Amk-control	0.0142^∗^	0.432^ns^	0.0144^∗^	0.0052^∗∗^	0.0902^ns^	0.0041^∗∗^
	Cur-control	0.25^ns^	0.001^∗∗^	0.5814^ns^	< 0.0001^∗∗∗∗^	0.6787^ns^	0.0027^∗∗^
	C3-control	0.0048^∗∗^	< 0.0001^∗∗∗∗^	0.0010^∗∗∗^	< 0.0001^∗∗∗∗^	0.0015^∗∗^	< 0.0001^∗∗∗∗^
C4	Kan-control	0.1520^ns^	0.398^ns^	0.0378^∗^	< 0.0001^∗∗∗∗^	0.1705^ns^	0.0069^∗∗^
	Cur-control	0.47^ns^	0.0809^ns^	0.3709^ns^	< 0.0001^∗∗∗∗^	0.1029^ns^	0.0020^∗∗^
	C4-control	0.0132^∗^	0.0054^∗∗^	0.0036^∗∗^	< 0.0001^∗∗∗∗^	0.0023^∗∗^	< 0.0001^∗∗∗∗^
C5	Amx-control	0.3528^ns^	0.2173^ns^	0.1184^ns^	0.0676^ns^	0.0703^ns^	0.0454^∗^
	Cur-control	0.3307^ns^	0.1312^ns^	0.1826^ns^	0.0048^∗∗^	0.5775^ns^	0.0026^∗∗^
	C5-control	0.0601^ns^	0.0070^∗∗^	0.0144^∗^	0.0003^∗∗∗^	0.0040^∗∗^	0.0003^∗∗∗^

*Note:* C1: combination of cefazolin and thymol; C2: combination of amikacin and curcumin; C3: combination of cefixime and thymol; C4: combination of kanamycin and curcumin; C5: combination of amoxicillin and curcumin. Control: biofilm from untreated wells; ns: nonsignificant correlation; Amk: amikacin, Amx: amoxicillin, Cfz: cefazolin, Cfx: cefixime, Kan: kanamycin, Cur: curcumin, Thy: thymol. Differences were considered significant at ^∗^*p* < 0.05, ^∗∗^*p* < 0.01, ^∗∗∗^*p* < 0.001, ^∗∗∗∗^*p* < 0.0001.

^∗^Significant correlation.

**Table 7:** IC50 values of antibiotics and natural products on UROtsa and Raw 264.7 cell lines.

Antimicrobial agents	IC50 values (µg/mL)	
	UROtsa	Raw 264.7
Amoxicillin	—	—
Amikacin	—	—
Cefixime	—	473.83
Kanamycin	—	—
Cefazolin	—	504.26
Thymol	—	441.78
Curcumin	51.89	94.40
Doxorubicin	1.58	1.26

*Note:* —: IC50 > 1000 µg/mL.

## Data Availability

Data are available on request from the authors.

## Supporting Information

Supporting information S1–S9 provide data on the additive/indifferent interaction of the combination of antibiotics and natural products against planktonic cells, biofilm inhibition, and biofilm eradication of *Staphylococcus aureus*, *Staphylococcus saprophyticus*, and *Staphylococcus epidermidis*.

S1: Additive/indifferent interaction of the combination of antibiotics and natural products against planktonic cells of *Staphylococcus aureus*.

S2: Additive/indifferent interaction of the combination of antibiotics and natural products against planktonic cells of *Staphylococcus saprophyticus*.

S3: Additive/indifferent interaction of the combination of antibiotics and natural products against planktonic cells of *Staphylococcus epidermidis*.

S4: Additive/indifferent interaction of antibiotic and natural product combinations on *Staphylococcus aureus* biofilm inhibition.

S5: Additive/indifferent interaction of antibiotic and natural product combinations on *Staphylococcus saprophyticus* biofilm inhibition.

S6: Additive/indifferent interaction of antibiotic and natural product combinations on *Staphylococcus epidermidis* biofilm inhibition.

S7: Additive/indifferent interaction of antibiotic and natural product combinations on *Staphylococcus aureus* biofilm eradication.

S8: Additive/indifferent interaction of antibiotic and natural product combinations on *Staphylococcus saprophyticus* biofilm eradication.

S9: Additive/indifferent interaction of antibiotic and natural product combinations on *Staphylococcus epidermidis* biofilm eradication.
