# Supplementary material for: Combining Plant Bioactives With Antibiotics for Enhanced Antibiofilm Activity Against Uropathogenic Staphylococcus spp. and Cytotoxicity Evaluation
Source: Adv Pharmacol Pharm Sci. 2025 Aug 4;2025:7461209. doi: 10.1155/adpp/7461209 (PMC12339163; doi:10.1155/adpp/7461209)
Supplement: Supporting Information — Additional supporting information can be found online in the Supporting Information section. [file 7461209.f1.docx]

**Combining Plant Bioactives with Antibiotics for Enhanced Anti-Biofilm Activity Against Uropathogenic *Staphylococcus spp.* and Cytotoxicity Evaluation**

Ulrich Joël Tsopmene^1^, Christian Ramsès Kuate Tokam^2^, Larissa Yetendje Chimi^1^, Nathalie Boulens^3,4^, Eric Allémann^3,4^, Florence Delie^3,4^, Clautilde Teugwa Mofor^5^, Jean Paul Dzoyem^1,3,4^*

^1^Department of Biochemistry, Faculty of Science, University of Dschang, Dschang, Cameroon.

^2^Department of Pharmaceutical Sciences, Faculty of Medicine and Pharmaceutical Sciences,

University of Dschang, Dschang, Cameroon.

^3^School of Pharmaceutical Sciences, University of Geneva, Geneva, Switzerland.

^4^Institute of Pharmaceutical Sciences of Western Switzerland, University of Geneva, Geneva, Switzerland.

^5^Department of Biochemistry, Faculty of Science, Laboratory of Phytobiochemistry and Medicinal Plants Studies, University of Yaoundé I, PO Box 812, Yaoundé.

*****Correspondence : Jean Paul Dzoyem, E.mail: [jpdzoyem@yahoo.fr](mailto:jpdzoyem@yahoo.fr) Tel: +237676091031, and Clautilde Teugwa Mofor E.mail: [moforclautilde@gmail.com](mailto:moforclautilde@gmail.com) Tel: +237675038292.

**Supporting Information**

Supplementary information S1-S9, provide data of Additive/indifferent interaction of the combination of antibiotics and natural products against planktonic cells, biofilm inhibition and biofilm eradication of *Staphylococcus aureus*, *Staphylococcus saprophyticus* and *Staphylococcus epidermidis*.

**S1. Additive/indifferent interaction of the combination of antibiotics and natural products against planktonic cells of Staphylococcus aureus.**

| **Antimicrobial agent in combination (*S. aureus*)** | **MIC (µg/mL)** | | | |  | |  |  |
| --- | --- | --- | --- | --- | --- | --- | --- | --- |
|  | **Alone** | | **Combined** | | **FIC** | | **MIC reduction fold of ATB** | FICI/ Interpretation |
|  | **ATB** | **NPs** | **ATB** | **NPs** | **ATB** | **NPs** |  |  |
| Amk+ Cur | 4.33 | 234.66 | 1.75 | 186.6 | 0.40 | 0.79 | 2.47 | 1.19/Ind |
| Amk+Plu | 4.33 | 6 | 0.875 | 3.66 | 0.20 | 0.61 | 4.95 | 0.81/Add |
| Amk+Ber | 4.33 | 138.66 | 2 | 117.3 | 0.46 | 0.84 | 2.16 | 1.30/ Ind |
| Amk+ Thy | 4.33 | 298.66 | 2.20 | 85.33 | 0.50 | 0.28 | 1.96 | 0.79/ Add |
| Amk+Que | 4.33 | 341.33 | 3 | 341.3 | 0.69 | 1 | 1.44 | 1.69/ Ind |
| Amk+Gal | 4.33 | 426.66 | 4.33 | 426.6 | 1 | 1 | 1 | 2/Ind |
| Dox+Cur | 6.16 | 234.66 | 2.83 | 109.3 | 0.45 | 0.46 | 2.17 | 0.92/Add |
| Dox+Plu | 6.16 | 4.66 | 0.35 | 3.33 | 0.05 | 0.71 | 17.6 | 0.77/ Add |
| Dox+ Ber | 6.16 | 138.66 | 5 | 96 | 0.81 | 0.69 | 1.23 | 1.50/ Ind |
| Dox+ Thy | 6.16 | 298.66 | 0.79 | 184 | 0.12 | 0.61 | 7.79 | 0.74/ Add |
| Dox+ Que | 6.16 | 426.66 | 5.66 | 384 | 0.91 | 0.9 | 1.08 | 1.02/ Ind |
| Dox+Gal | 6.16 | 426.66 | 6.02 | 261.3 | 0.97 | 0.61 | 1.02 | 1.58/ Ind |
| Cfz+Cur | 42.66 | 242.30 | 18.11 | 55.42 | 0.42 | 0.22 | 2.35 | 0.65/ Add |
| Cfz +Plu | 42.66 | 4.66 | 32.33 | 4.33 | 0.75 | 0.92 | 1.31 | 1.68/ Ind |
| Cfz +Ber | 42.66 | 138.66 | 32 | 109.3 | 0.75 | 0.78 | 1.33 | 1.53/ Ind |
| Cfz +Thy | 42.66 | 298.66 | 13.33 | 277.3 | 0.31 | 0.92 | 3.2 | 1.24/ Ind |
| Cfz +Que | 42.66 | 341.33 | 42.66 | 341.3 | 1 | 1 | 1 | 2/ Ind |
| Cfz +Gal | 42.66 | 426.66 | 42.66 | 426.6 | 1 | 1 | 1 | 2/ Ind |
| Amx+Cur | 86.66 | 234.66 | 48.66 | 64 | 0.56 | 0.27 | 1.78 | 0.83/ Add |
| Amx+Plu | 86.66 | 4.66 | 64.66 | 3 | 0.74 | 0.64 | 1.34 | 1.38/ Ind |
| Amx+ Ber | 86.66 | 138.66 | 64.66 | 138.6 | 0.74 | 1 | 1.34 | 1.74/ Ind |
| Amx+Thy | 86.66 | 298.66 | 64.66 | 234.6 | 0.74 | 0.78 | 1.34 | 1.53/ Ind |
| Amx+Que | 86.66 | 341.33 | 86.66 | 341.3 | 1 | 1 | 1 | 2/ Ind |
| Amx+Gal | 86.66 | 426.67 | 86.66 | 426.6 | 0.99 | 1 | 1 | 1.99/ Ind |
| Kan+Cur | 22.66 | 149.33 | 9.33 | 37.33 | 0.41 | 0.25 | 2.42 | 0.66/ Add |
| Kan+Pb | 22.66 | 4.66 | 11.66 | 2.16 | 0.51 | 0.46 | 1.95 | 0.97/ Add |
| Kan+Ber | 22.66 | 96 | 22.66 | 74.66 | 1 | 0.77 | 1 | 1.77/ Ind |
| Kan+Thy | 22.66 | 298.66 | 13.33 | 213.3 | 0.58 | 0.71 | 1.69 | 1.30/ Ind |
| Kan+Que | 22.66 | 341 .3 | 17.33 | 341.3 | 0.76 | 1 | 1.30 | 1.74/ Ind |
| Kan+Gal | 22.66 | 426.66 | 17.33 | 341.33 | 0.76 | 0.8 | 1.30 | 0.95/ Add |
| Ery+Cur | 108 | 234.66 | 43 | 149.33 | 0.39 | 0.63 | 2.51 | 1.03/ Ind |
| Ery+Plu | 108 | 4.66 | 43 | 3.66 | 0.39 | 0.78 | 2.51 | 1.18/ Ind |
| Ery+Ber | 108 | 138.67 | 107.33 | 74.66 | 0.99 | 0.53 | 1 | 1.53/ Ind |
| Ery+Thy | 108 | 298.66 | 108 | 256 | 1 | 0.85 | 1 | 1.85/ Ind |
| Ery+Que | 108 | 341.33 | 108 | 341.33 | 1 | 1 | 1 | 2/ Ind |
| Ery+Gal | 108 | 426.66 | 43.33 | 298.66 | 0.40 | 0.7 | 2.49 | 1.10/ Ind |
| Cfx+Cur | 213.33 | 234.66 | 213.33 | 234.66 | 1 | 1 | 1 | 2/ Ind |
| Cfx+Plu | 213.33 | 4.66 | 213.33 | 4.66 | 1 | 0.85 | 1 | 1.85/ Ind |
| Cfx+Ber | 213.33 | 138.66 | 213.33 | 138.66 | 1 | 1 | 1 | 2/ Ind |
| Cfx+Thy | 213.33 | 341.33 | 192 | 213.33 | 0.9 | 0.625 | 1.11 | 1.52/ Ind |
| Cfx+Que | 213.33 | 341.33 | 213.33 | 341.33 | 1 | 1 | 1 | 2/ Ind |
| Cfx+Gal | 213.33 | 426.66 | 213.33 | 426.66 | 1 | 1 | 1 | 2/ Ind |

MIC: mean of the minimum inhibitory concentrations; FIC: fractional inhibitory concentrations; FICI: fractional inhibitory index (FICI); NPs: natural products; Add: additivity; Ind: indifference; AK: amikacin; Dox: doxycycline; Cfx: cefazolin; Amx: amoxicillin; Kan: kanamycin; Ery: erythromycin; Cfx: cefixime; Cur: curcumin; Plu: plumbagin; Ber: berberine; Thy: thymol; Que: quercetin; Gal: gallic acid.

**S2**. **Additive/indifferent interaction of the combination of antibiotics and natural products against planktonic cells of Staphylococcus saprophyticus.**

| **Antimicrobial agent in combination (*S. saprohyticus*)** | **MIC (µg/mL)** | | | |  | |  |  |
| --- | --- | --- | --- | --- | --- | --- | --- | --- |
|  | **Alone** | | **Combined** | | **FIC** | | MIC reduction fold of ATB | FICI/ Interpretation |
|  | **ATB** | **NPs** | **ATB** | **NPs** | **ATB** | **NPs** |  |  |
| Amk+ Cur | 22 | 170.66 | 3 | 69.33 | 0.13 | 0.40 | 7.33 | 0.54/Add |
| Amk+Plu | 22 | 8.66 | 16.04 | 6 | 0.72 | 0.69 | 1.37 | 1.4/Ind |
| Amk+Ber | 22 | 149.33 | 16.3 | 138.66 | 0.74 | 0.92 | 1.6 | 1.34/ Ind |
| Amk+ Thy | 22 | 341.33 | 14 | 277.33 | 0.63 | 0.8 | 1.44 | 1.57/ Ind |
| Amk+Que | 22 | 341.33 | 21.41 | 298.66 | 0.97 | 0.87 | 1.84 | 1.02/ Ind |
| Amk+ Gal | 22 | 426.66 | 21.41 | 384 | 0.97 | 0.9 | 1.87 | 1.02/ Ind |
| Dox+Cur | 32.16 | 149.33 | 13.41 | 46.66 | 0.41 | 0.31 | 2.39 | 0.72/ Add |
| Dox+Plu | 32.16 | 8.66 | 4.0 | 4,33 | 0.12 | 0.5 | 7.42 | 0.62/ Add |
| Dox+ Ber | 32.16 | 213.33 | 32.04 | 149.33 | 0.99 | 0.7 | 1.00 | 1.69/ Ind |
| Dox+ Thy | 32.16 | 341.3 | 26.67 | 341.33 | 0.8 | 1 | 1.20 | 1.82/ Ind |
| Dox+ Que | 32.1 | 426.66 | 21.5 | 362.66 | 0.66 | 0.85 | 1.49 | 1.51/ Ind |
| Dox+Gal | 32,16 | 426.66 | 16.04 | 234.66 | 0.49 | 0.55 | 2.00 | 1.04/ Ind |
| Cfz+Cur | 64.66 | 213.33 | 34 | 74.66 | 0.52 | 0.35 | 1.90 | 0.87/Add |
| Cfz+Plu | 64.66 | 8.33 | 16.16 | 5.5 | 0.25 | 0.66 | 4 | 0.91/Add |
| Cfz+Ber | 64.66 | 213.33 | 27.33 | 170.66 | 0.42 | 0.8 | 2.36 | 1.22/ Ind |
| Cfz+Thy | 64.66 | 341.33 | 43 | 213.33 | 0.66 | 0.62 | 1.50 | 1.28/ Ind |
| Cfz+Que | 64.66 | 426.66 | 45.66 | 384 | 0.70 | 0.9 | 1.41 | 1.60/ Ind |
| Cfz+Gal | 64.66 | 426.66 | 32.66 | 362.66 | 0.50 | 0.85 | 1.97 | 1.35/ Ind |
| Amx+Cur | 22 | 149.33 | 14 | 85.33 | 0.63 | 0.57 | 1.57 | 1.20/ Ind |
| Amx+Plu | 22 | 8.33 | 16.08 | 3.33 | 0.73 | 0,4 | 1.36 | 1.13/ Ind |
| Amx+ Ber | 22 | 213.33 | 4.66 | 133.33 | 0.21 | 0.62 | 4.71 | 0.83/Add |
| Amx+Thy | 22 | 341.33 | 22 | 341.33 | 1 | 1 | 1 | 2/ Ind |
| Amx+Que | 22 | 341.33 | 16.66 | 266.66 | 0.75 | 0.78 | 1.32 | 1.53/ Ind |
| Amx+Gal | 22 | 426.66 | 14 | 352 | 0.63 | 0.82 | 1.57 | 1.46/ Ind |
| Kan+Cur | 14 | 213.33 | 13.66 | 138.66 | 0.97 | 0.65 | 1.02 | 1.62/ Ind |
| Kan+Plu | 14 | 8.33 | 3.35 | 4.166 | 0.23 | 0.5 | 4.17 | 0.73/Add |
| Kan+Ber | 14 | 213.3 | 12 | 192 | 0.85 | 0.9 | 1.16 | 1.75/Ind |
| Kan+Thy | 14 | 341.33 | 12.33 | 170.66 | 0.88 | 0.5 | 1.13 | 1.38/ Ind |
| Kan+Que | 14 | 426.66 | 12 | 346.66 | 0.85 | 0.81 | 1.16 | 1.66/ Ind |
| Kan+Gal | 14 | 426.66 | 6 | 298.66 | 0.42 | 0.7 | 2.33 | 1.12/ Ind |
| Ery+Cur | 4.66 | 149.33 | 2.04 | 53.33 | 0.43 | 0.35 | 2.28 | 0.79/Add |
| Ery+Plu | 4.66 | 8.33 | 1.83 | 3.33 | 0.39 | 0.4 | 2.54 | 0.79/Add |
| Ery+Ber | 4.66 | 298.66 | 4.33 | 258.66 | 0.9 | 0.86 | 1.07 | 1.79/ Ind |
| Ery+Thy | 4.66 | 341.33 | 4.66 | 341.33 | 1 | 1 | 1 | 2/ Ind |
| Ery+Que | 4.66 | 341.33 | 4.08 | 266.66 | 0.87 | 0.78 | 1.14 | 1.65/ Ind |
| Ery+Gal | 4.66 | 426.66 | 2.33 | 224 | 0.5 | 0.52 | 2 | 1.02/ Ind |
| Cfx+Cur | 192 | 213.33 | 192 | 149.33 | 1 | 0.7 | 1 | 1.7/ Ind |
| Cfx+Plu | 192 | 8.33 | 192 | 8.16 | 1 | 0.98 | 1 | 1.98/ Ind |
| Cfx+Ber | 192 | 298.66 | 192 | 298.66 | 1 | 1 | 1 | 2/ Ind |
| Cfx+Thy | 128 | 341.33 | 128 | 256 | 1 | 075 | 1 | 1.75/ Ind |
| Cfx+Que | 192 | 341.33 | 192 | 341.33 | 1 | 1 | 1 | 2/ Ind |
| Cfx+Gal | 192 | 341.33 | 192 | 341.33 | 1 | 1 | 1 | 2/ Ind |

MIC: Mean of the minimum inhibitory concentrations; FIC: fractional inhibitory concentrations; FICI: fractional inhibitory index (FICI); NPs: natural products; Add: additivity; Ind: indifference; Ami: amikacin; Dox: doxycycline; Cfz: cefazolin; Amx: amoxicillin; Kan: kanamycin; Ery: erythromycin; Cfx: cefixime; Cur: curcumin; Plu: plumbagin; Ber: berberine; Thy: thymol; Que: quercetin; Gal: gallic acid.

**S3**. **Additive/indifferent interaction of the combination of antibiotics and natural products against planktonic cells of Staphylococcus epidermidis.**

| **Antimicrobial agent in combination**  **(*S. epidermidis*)** | **Alone** | | **Combined** | | **FIC** | | **MIC reduction fold of ATB** | FICI/ Interpretation |
| --- | --- | --- | --- | --- | --- | --- | --- | --- |
|  | **ATB** | **NPs** | **ATB** | **NPs** | **ATB** | **NPs** |  |  |
| Amk+Plu | 1.16 | 6.66 | 1.16 | 6 | 1 | 0.9 | 1 | 1.9/Ind |
| Amk+Ber | 1.16 | 213.33 | 1.16 | 6 | 1 | 0.02 | 1 | 1.02/Ind |
| Amk+ Thy | 1.16 | 426.66 | 0.52 | 96 | 0.44 | 0.225 | 2.24 | 0.67/Add |
| Amk+Que | 1.16 | 512 | 1 | 426.66 | 0.85 | 0.83 | 1.16 | 1.69/Ind |
| Amk+Gal | 1.16 | 426.66 | 0.83 | 341.33 | 0.71 | 0.8 | 1.4 | 1.51/Ind |
| Dox+Cur | 7 | 384 | 2.04 | 213.33 | 0.29 | 0.55 | 3.42 | 0.84/Add |
| Dox+Plu | 7 | 6.66 | 2.16 | 3.33 | 0.30 | 0.5 | 3.23 | 0.80/Add |
| Dox+ Ber | 7 | 213.33 | 2.08 | 128 | 0.29 | 0.6 | 3.36 | 0.89/Add |
| Dox+ Que | 7 | 512 | 4.08 | 277.33 | 0.58 | 0.54 | 1.71 | 1.12/Ind |
| Cfz+Cur | 42.66 | 384 | 21.33 | 202.66 | 0.5 | 0.52 | 2 | 1.02/Ind |
| Cfz+Plu | 42.66 | 6.66 | 16 | 2.33 | 0.37 | 0.35 | 2.66 | 0.72/Add |
| Cfz+Ber | 42.66 | 21333 | 29.33 | 138.66 | 0.68 | 0.65 | 1.45 | 1.33/Ind |
| Cfz+Thy | 42.66 | 426.66 | 28 | 256 | 0.65 | 0.6 | 1.52 | 1.25/Ind |
| Cfz+Que | 42.66 | 512 | 37.33 | 512 | 0.87 | 1 | 1.14 | 1.87/Ind |
| Cfz+Gal | 42.66 | 426.66 | 33.33 | 341.33 | 0.78 | 0.8 | 1.28 | 1.58/Ind |
| Amx+Cur | 26.83 | 384 | 16.041 | 256 | 0.59 | 0.66 | 1.67 | 1.26/Ind |
| Amx+Plu | 26.83 | 6.66 | 26.83 | 4.66 | 1 | 0.7 | 1 | 1.7/Ind |
| Amx+ Ber | 26.83 | 213.33 | 8.041 | 88 | 0.29 | 0.41 | 3.33 | 0.71/Ind |
| Amx+Thy | 26.83 | 426.66 | 11.37 | 170.66 | 0.42 | 0.4 | 2.35 | 0.82/Add |
| Amx+Que | 26.83 | 512 | 6.83 | 256 | 0.25 | 0.5 | 3.92 | 0.75/Add |
| Amx+Gal | 26.83 | 512 | 6.83 | 256 | 0.25 | 0.5 | 3.92 | 0.75/Add |
| Kan+Cur | 7.33 | 384 | 5.5 | 202.66 | 0.75 | 0.52 | 1.33 | 1.27/Ind |
| Kan+Plu | 7.33 | 6.66 | 5.58 | 4 | 0.76 | 0.6 | 1,31 | 1.36/Ind |
| Kan+Ber | 7.33 | 298.66 | 2.08 | 128 | 0.28 | 0.42 | 3.52 | 0.71/Ind |
| Kan+Thy | 7.33 | 426.66 | 5.42 | 224 | 0.74 | 0.52 | 1.35 | 1.26/Ind |
| Kan+Que | 7.33 | 512 | 7 | 512 | 0.95 | 1 | 1.04 | 1.95/Ind |
| Kan+Gal | 7.33 | 426.66 | 7 | 426.66 | 0.95 | 1 | 1.04 | 1.95/Ind |
| Ery+Cur | 32.66 | 384 | 21.41 | 213.33 | 0.65 | 0.55 | 1.52 | 1.21/Ind |
| Ery+Plu | 32.66 | 6.66 | 6.33 | 4.66 | 0.19 | 0.7 | 5.15 | 0.89/Add |
| Ery+Ber | 32.66 | 213.33 | 24 | 128 | 0.73 | 0.6 | 1.36 | 1.33/Ind |
| Ery+Thy | 32.66 | 426.66 | 8.33 | 213.33 | 0.25 | 0.5 | 3.92 | 0.75/Add |
| Ery+Que | 32.66 | 512 | 32.66 | 512 | 1 | 1 | 1 | 2/Ind |
| Ery+Gal | 32.66 | 426.66 | 4 | 256 | 0.12 | 0.6 | 8.16 | 0.72/Add |
| Cfx+Cur | 106.66 | 384 | 106.66 | 384 | 1 | 1 | 1 | 2/Ind |
| Cfx+Plu | 106.66 | 25.33 | 69.3 | 25.33 | 0.65 | 1 | 1.53 | 1.65/Ind |
| Cfx+Ber | 106.66 | 213.33 | 106.66 | 192 | 1 | 0.9 | 1 | 1.9/Ind |
| Cfx+Thy | 106.66 | 426.66 | 106.66 | 426.66 | 1 | 1 | 1 | 2/Ind |
| Cfx+Que | 106.66 | 362.66 | 106.66 | 362.66 | 1 | 1 | 1 | 2/Ind |
| Cfx+Gal | 106.66 | 277.33 | 106.66 | 341.33 | 1 | 1.23 | 1.23 | 2.23/Ind |

MIC: Mean of the minimum inhibitory concentrations; FIC: fractional inhibitory concentrations; FICI: fractional inhibitory index (FICI); NPs: natural products; Add: additivity; Ind: indifference; Amk: amikacin; Dox: doxycycline; Cfz: cefazolin; Amx: amoxicillin; Kan: kanamycin; Ery: erythromycin; Cfx: cefixime; Cur: curcumin; Plu: plumbagin; Ber: berberine; Thy: thymol; Que: quercetin; Gal: gallic acid.

**S4**. **Additive/indifferent interaction of antibiotic and natural product combinations on Staphylococcus aureus biofilm inhibition.**

| **Antimicrobial agent in combination (*S. aureus*)** | **MBIC (µg/mL)** | | | |  | |  |  |
| --- | --- | --- | --- | --- | --- | --- | --- | --- |
|  | **Alone** | | **Combined** | | **FIC** | | **MIC reduction fold of ATB** | FICI/Interpretation |
|  | **ATB** | **NPs** | **ATB** | **NPs** | **ATB** | **NPs** |  |  |
| Amk+ Cur | 1.83 | 106.66 | 0.52 | 37.33 | 0.28 | 1.83 | 3.52 | 0.63/Add |
| Amk+Plu | 1.83 | 10.66 | 0.37 | 4.66 | 0.20 | 0.43 | 4.88 | 0.64/Add |
| Amk+Ber | 1.83 | 170.66 | 1.58 | 128 | 0.86 | 0.75 | 1.15 | 1.61/Ind |
| Amk+Que | 1.83 | 512 | 1.70 | 346.66 | 0.93 | 0.67 | 1.07 | 1.60/Ind |
| Amk+Gal | 1.83 | 512 | 1.83 | 512 | 1 | 1 | 1 | 2/Ind |
| Dox+Cur | 2.16 | 106.66 | 1.58 | 53.33 | 0.73 | 0.5 | 1.36 | 1.23/Ind |
| Dox+Plu | 2.16 | 90.66 | 0.58 | 33.33 | 0.26 | 0.36 | 3.71 | 0.63/Add |
| Dox+ Ber | 2.16 | 170.66 | 1.5 | 106.66 | 0.69 | 0.62 | 1.44 | 1.31/Ind |
| Dox+ Thy | 2.16 | 341.33 | 0.70 | 74.66 | 0.32 | 0.21 | 3.05 | 0.54/Add |
| Dox+ Que | 2.16 | 426.66 | 2.16 | 426.66 | 1 | 1 | 1 | 2/Int |
| Dox+Gal | 2.16 | 512 | 0.83 | 234.66 | 0.38 | 0.45 | 2.6 | 0.84/Add |
| Cfz+Cur | 4.66 | 234.66 | 4.33 | 197.33 | 0.92 | 0.84 | 1.07 | 1.07/Ind |
| Cfz+Ber | 4.66 | 170.66 | 1.16 | 45.33 | 0.25 | 0.26 | 4 | 0.51/Add |
| Cfz+Thy | 4.66 | 341.33 | 4.04 | 213.33 | 0.86 | 0.625 | 1.15 | 1.49/Ind |
| Cfz+Que | 4.66 | 512 | 2.33 | 218.66 | 0.5 | 0.42 | 2 | 0.92/Add |
| Cfz+Gal | 4.66 | 512 | 3.33 | 384 | 0.71 | 0.75 | 1.4 | 1.46/Ind |
| Amx+Cur | 1.5 | 106.66 | 0.83 | 74.66 | 0.55 | 0.7 | 1.8 | 1.25/Ind |
| Amx+Plu | 1.5 | 9.33 | 0.87 | 6.66 | 0.58 | 0.71 | 1.71 | 1.29/Ind |
| Amx+ Ber | 1.5 | 170.66 | 1.5 | 74.66 | 1 | 0.43 | 1 | 1.43/Ind |
| Amx+Thy | 1.5 | 341.33 | 1.08 | 149.33 | 0.72 | 0.43 | 1.38 | 1.15/Ind |
| Amx+Que | 1.5 | 512 | 1.5 | 512 | 1 | 1 | 1 | 2/Ind |
| Amx+Gal | 1.5 | 512 | 1.41 | 426.66 | 0.94 | 0.83 | 1.05 | 1.77/Ind |
| Kan+Cur | 1.83 | 234.66 | 1.43 | 186.66 | 0.78 | 0.79 | 1.27 | 1.57/Ind |
| Kan+Pb | 1.83 | 10.66 | 0.33 | 5.33 | 0.18 | 0.5 | 5.5 | 0.68/Add |
| Kan+Ber | 1.83 | 170.66 | 0.91 | 42.66 | 0.5 | 0.25 | 2 | 0.75/Add |
| Kan+Que | 1.83 | 512 | 1.68 | 384 | 0.92 | 0.75 | 1.08 | 1.67/Ind |
| Kan+Gal | 1.83 | 512 | 1.5 | 216 | 0.81 | 0.42 | 1.22 | 1.24/Ind |
| Ery+Cur | 42.83 | 106.66 | 24.08 | 53.33 | 0.56 | 0.5 | 1.77 | 1.06/Ind |
| Ery+Plu | 42.83 | 13.33 | 26.68 | 6 | 0.62 | 0.45 | 1.60 | 1.07/Ind |
| Ery+Ber | 42.83 | 170.66 | 42.75 | 128 | 0.99 | 0.75 | 1.00 | 1.74/Ind |
| Ery+Thy | 42.83 | 341.33 | 42.70 | 181.33 | 0.99 | 0.53 | 1.00 | 1.52/Ind |
| Ery+Que | 42.83 | 362.66 | 42.83 | 362.66 | 1 | 1 | 0.22 | 2/Ind |
| Ery+Gal | 42.83 | 512 | 42.83 | 512 | 1 | 1 | 1 | 2/Ind |
| Cfx+Cur | 64 | 106.66 | 64 | 106.66 | 1 | 1 | 1 | 2/Ind |
| Cfx+Plu | 64 | 10.66 | 45.33 | 6,66 | 0.70 | 0.62 | 1.41 | 1.33/Ind |
| Cfx+Ber | 64 | 170.66 | 48 | 128 | 0.75 | 0.75 | 1.33 | 1.5/Ind |
| Cfx+Thy | 64 | 341.33 | 64 | 192 | 1 | 0.56 | 1 | 1.56/Ind |
| Cfx+Que | 64 | 512 | 64 | 512 | 1 | 1 | 1 | 2/Ind |
| Cfx+Gal | 64 | 512 | 64 | 512 | 1 | 1 | 1 | 2/Ind |

MIC: Mean of the minimum biofilm inhibitory concentrations; FIC: fractional inhibitory concentrations; FICI: fractional inhibitory index (FICI); NPs: natural products; Add: additivity; Ind: indifference; Amk: amikacin; Dox: doxycycline; Cfz: cefazolin; Amx: amoxicillin; Kan: kanamycin; Ery: erythromycin; Cfx: cefixim; Cur: curcumin; Pb: plumbagin; Ber: berberine; Thy: thymol; Que: quercetin; Gal: gallic acid.

**S5. Additive/indifferent interaction of antibiotic and natural product combinations on Staphylococcus saprophyticus biofilm inhibition.**

| Antimicrobial agent in combination (*S. saprophyticcus*) | MBIC (µg/mL) | | | |  | |  |  |
| --- | --- | --- | --- | --- | --- | --- | --- | --- |
|  | Alone | | Combined | | FIC | | MIC reduction fold of ATB | FICI/Interpretation |
|  | ATB | NPs | ATB | NPs | ATB | NPs |  |  |
| Amk+Plu | 2.08 | 6.66 | 1.70 | 3.33 | 0.82 | 0.5 | 2,04 | 0.64/Add |
| Amk+Ber | 2.08 | 106.66 | 1.02 | 16 | 0.49 | 0.15 | 1.09 | 0.91/Add |
| Amk+Que | 2.08 | 426.66 | 2.02 | 352 | 0.97 | 0.82 | 1.03 | 1.79/Int |
| Amk+Gal | 2.08 | 426.66 | 2.08 | 426.66 | 1 | 1 | 1 | 2/Ind |
| Dox+Cur | 0.5 | 106.66 | 0.38 | 26.66 | 0.77 | 0.25 | 1.29 | 1.02/Ind |
| Dox+ Ber | 0.5 | 74.66 | 0.21 | 24.04 | 0.43 | 0.32 | 2.27 | 0.76/Add |
| Dox+ Que | 0.5 | 426.66 | 0.45 | 352 | 0.91 | 0.82 | 1.09 | 0.91/Add |
| Dox+Gal | 0.5 | 426.66 | 0.45 | 384 | 0.91 | 0.9 | 1.09 | 1.81/Ind |
| Cfz+Plu | 8.33 | 6.66 | 2.87 | 4 | 0.34 | 0.6 | 2.89 | 0.94/Add |
| Cfz+Ber | 8.33 | 85.33 | 5.83 | 32 | 0.7 | 0.37 | 1.42 | 1.07/Ind |
| Cfz+Que | 8.33 | 426.66 | 6.33 | 426.66 | 0.76 | 1 | 1.31 | 1.76/Ind |
| Cfz+Gal | 8.33 | 426.66 | 6.33 | 384 | 0.76 | 0.9 | 1.31 | 1.66/Ind |
| Amx+Plu | 8.33 | 6.66 | 1.75 | 2.33 | 0.21 | 0.35 | 4.76 | 0.56/Add |
| Amx+Thy | 8.33 | 128 | 0.33 | 64 | 0.04 | 0.5 | 25 | 0.54/Add |
| Amx+Que | 8.33 | 426.66 | 8.08 | 362.66 | 0.97 | 0.85 | 1.03 | 1.82/Ind |
| Amx+Gal | 8.33 | 426.66 | 8.08 | 352 | 0.97 | 0.82 | 1.03 | 1.79/Ind |
| Kan+Plu | 4.083 | 6.66 | 1.45 | 2.33 | 0.35 | 0.35 | 2.8 | 0.70/Add |
| Kan+Ber | 4.083 | 85.33 | 1.75 | 74.66 | 0.42 | 0.87 | 2.33 | 1.30/Ind |
| Kan+Thy | 4.083 | 128 | 1.70 | 21.33 | 0.41 | 0.16 | 2.39 | 0.58/Add |
| Kan+Que | 4.083 | 426.66 | 4.08 | 426.66 | 1 | 1 | 1 | 2/Ind |
| Kan+Gal | 4.083 | 426.66 | 4.08 | 426.66 | 1 | 1 | 1 | 2/Ind |
| Ery+Cur | 27.33 | 6.66 | 21.4 | 3.66 | 0.78 | 0.55 | 1.27 | 1.33/Ind |
| Ery+Plu | 27.33 | 6.66 | 21.43 | 3.66 | 0.78 | 0.55 | 1.27 | 1.33/Ind |
| Ery+Ber | 27.33 | 85.33 | 27.33 | 64 | 1 | 0.75 | 1 | 1.75/Ind |
| Ery+Thy | 27.33 | 128 | 24.66 | 128 | 0.90 | 1 | 1.10 | 1.9/Ind |
| Ery+Que | 27.33 | 512 | 6.08 | 512 | 0.22 | 1 | 4.49 | 1,22/Ind |
| Ery+Gal | 27.33 | 426.66 | 27.33 | 426.6 | 1 | 1 | 1 | 2/Ind |
| Cfx+Cur | 48 | 106.66 | 45.33 | 69.33 | 0.94 | 0.65 | 1.05 | 1.59/Ind |
| Cfx+Plu | 48 | 6.66 | 42.75 | 4 | 0.89 | 0.6 | 1.12 | 1.49/Ind |
| Cfx+Ber | 48 | 106.66 | 42.75 | 74.66 | 0.89 | 0.7 | 1.12 | 1.59/Ind |
| Cfx+Thy | 48 | 128 | 43.33 | 112 | 0.90 | 0.87 | 1.10 | 1.77/Ind |
| Cfx+Quer | 48 | 426.66 | 42.83 | 256 | 0.89 | 0.6 | 1.12 | 1.49/Ind |
| Cfx+Gal | 48 | 426.66 | 43.33 | 362.6 | 0.90 | 0.85 | 1.10 | 1.75/Ind |

MBIC: Mean of the minimum biofilm inhibitory concentrations; FIC: fractional inhibitory concentrations; FICI: fractional inhibitory index (FICI); NPs: natural products; Add: additivity; Ind: indifference; Amk: amikacin; Dox: doxycycline; Cfz: cefazolin; Amx: amoxicillin; Kan: kanamycin; Ery: erythromycin; Cfx: cefixime; Cur: curcumin; Plu: plumbagin; Ber: berberine; Thy: thymol; Que: quercetin; Gal: gallic acid.

**S6. Additive/indifferent interaction of antibiotic and natural product combinations on Staphylococcus epidermidis biofilm inhibition.**

| **Antimicrobial agent in combination (*S. epidermidis*)** | **MBIC (µg/mL)** | | | |  | |  |  |
| --- | --- | --- | --- | --- | --- | --- | --- | --- |
|  | **Alone** | | **Combined** | | **FIC** | | **MIC reduction fold of ATB** | **FICI/Interpretation** |
|  | **ATB** | **NPs** | **ATB** | **NPs** | **ATB** | **NPs** |  |  |
| Amk+Plu | 3.08 | 8 | 0.12 | 4 | 0.04 | 0.5 | 24.66 | 0.54/Add |
| Amk+Ber | 3.08 | 256 | 2.87 | 98.66 | 0.93 | 0.38 | 1.07 | 1.31/Ind |
| Amk+ Thy | 3.08 | 213.33 | 2.79 | 106.66 | 0.90 | 0.5 | 1.10 | 1.40/Ind |
| Amk+Que | 3.08 | 512 | 3.08 | 512 | 1 | 1 | 1 | 2/Ind |
| Amk+Gal | 3.08 | 512 | 3.02 | 426.66 | 0.97 | 0.83 | 1.02 | 1.81/Ind |
| Dox+Cur | 3.08 | 128 | 0.33 | 69.33 | 0.10 | 0.54 | 9.25 | 0.64/Add |
| Dox+Plu | 3.08 | 8 | 1.43 | 1.66 | 0.46 | 0.20 | 2.14 | 0.67/Add |
| Dox+ Ber | 3.08 | 256 | 1.68 | 58.66 | 0.54 | 0.22 | 1.82 | 0.77/Add |
| Dox+ Thy | 3.41 | 213.33 | 1.68 | 26.66 | 0.49 | 0.125 | 2.02 | 0.61/Add |
| Dox+ Que | 3.08 | 512 | 3.02 | 346.66 | 0.97 | 0.67 | 1.02 | 1.65/Ind |
| Dox+Gal | 3.08 | 512 | 3.02 | 346.66 | 0.97 | 0.67 | 1.02 | 1.65/Add |
| Cfz+Plu | 8.66 | 8 | 2 | 3.33 | 0.23 | 0.41 | 4.33 | 0.64/Add |
| Cfz+Ber | 8.66 | 256 | 3.5 | 181.33 | 0.40 | 0.70 | 2.47 | 1.11/Ind |
| Cfz+Que | 8.66 | 512 | 8.66 | 512 | 1 | 1 | 1 | 2/Ind |
| Cfz+Ag | 8.66 | 512 | 8.66 | 512 | 1 | 1 | 1 | 2/Ind |
| Amx+ Ber | 2.08 | 256 | 1.37 | 138.66 | 0.66 | 0.54 | 1.51 | 1.20/Ind |
| Amx+Thy | 2.08 | 213.33 | 0.5 | 74.66 | 0.24 | 0.35 | 4.16 | 0.59/Add |
| Amx+Que | 2.08 | 512 | 2.08 | 384 | 1 | 0.75 | 1 | 1.75/Ind |
| Amx+Gal | 2.08 | 512 | 2.08 | 512 | 1 | 1 | 1 | 2/Ind |
| Kan+Cur | 24.66 | 128 | 24.08 | 96 | 0.97 | 0.75 | 1.02 | 1.72/Ind |
| Kan+Plu | 43.33 | 8 | 42.83 | 2.66 | 0.98 | 0.33 | 1.01 | 1.32/Ind |
| Kan+Thy | 24.66 | 213.33 | 24.16 | 138.66 | 0.97 | 0.65 | 1.02 | 1.62/Ind |
| Kan+Que | 24.66 | 384 | 24.66 | 384 | 1 | 1 | 1 | 2/Ind |
| Kan+Gal | 24.66 | 512 | 24.66 | 512 | 1 | 1 | 1 | 2/Ind |
| Ery+Cur | 24 | 128 | 12.02 | 74.66 | 0.50 | 0.58 | 1.99 | 1.08/Ind |
| Ery+Plu | 24 | 8 | 13.33 | 4.66 | 0.55 | 0.58 | 1.13 | 1.8/Ind |
| Ery+Ber | 24 | 256 | 18.66 | 181.33 | 0.77 | 0.70 | 1.28 | 1.48/Ind |
| Ery+Thy | 24 | 213.33 | 22.66 | 149.3 | 0.94 | 0.7 | 1.05 | 1.64/Ind |
| Ery+Que | 24 | 512 | 24 | 512 | 1 | 1 | 1 | 2/Ind |
| Ery+Gal | 24 | 512 | 24 | 512 | 1 | 1 | 1 | 2/Ind |
| Cfx+Cur | 64 | 128 | 64 | 128 | 1 | 1 | 1 | 2/Ind |
| Cfx+Plu | 64 | 8 | 64 | 8 | 1 | 1 | 1 | 2/Ind |
| Cfx+Ber | 64 | 256 | 64 | 256 | 1 | 1 | 1 | 2/Ind |
| Cfx+Thy | 64 | 213.33 | 64 | 213.33 | 1 | 1 | 1 | 2/Ind |
| Cfx+Que | 64 | 512 | 64 | 512 | 1 | 1 | 1 | 2/Ind |
| Cfx+Gal | 64 | 512 | 64 | 512 | 1 | 1 | 1 | 2/Ind |

MBIC: Mean of the minimum biofilm inhibitory concentrations; FIC: fractional inhibitory concentrations; FICI: fractional inhibitory index (FICI); NPs: natural products; Add: additivity; Ind: indifference; Amk: amikacin; Dox: doxycycline; Cfz: cefazolin; Amx: amoxicillin; Kan: kanamycin; Ery: erythromycin; Cfx: cefixime; Cur: curcumin; Plu: plumbagin; Ber: berberine; Thy: thymol; Que: quercetin; Gal: gallic acid.

**S7.** **Additive/indifferent interaction of antibiotic and natural product combinations on Staphylococcus aureus biofilm eradication.**

| **Antimicrobial agent in combination (*S. aureus*)** | **MBEC (µg/mL)** | | | |  | |  |  |
| --- | --- | --- | --- | --- | --- | --- | --- | --- |
|  | **Alone** | | **Combined** | | **FIC** | | **MIC reduction fold of ATB** | **FICI/Interpretation** |
|  | **ATB** | **NPs** | **ATB** | **NPs** | **ATB** | **NPs** |  |  |
| Amk+ Cur | 13.33 | 149.33 | 4 | 85.33 | 0.3 | 0.57 | 3.33 | 0.87/Add |
| Amk+Plu | 13.33 | 32 | 5.33 | 16 | 0.4 | 0.5 | 2.5 | 0.9/Add |
| Amk+ Thy | 13.33 | 106,66 | 4.33 | 37.33 | 0.32 | 0.35 | 3.07 | 0.67/Add |
| Amk+Que | 13.33 | 384 | 6.66 | 106.66 | 0.5 | 0.27 | 2 | 0.77/Add |
| Amk+Gal | 13.33 | 298.66 | 9.33 | 224 | 0.7 | 0.75 | 1.42 | 1.45/Ind |
| Dox+Cur | 16 | 128 | 6 | 48 | 0.37 | 0.37 | 2.66 | 0.75/Add |
| Dox+Plu | 16 | 16 | 2.75 | 6 | 0.17 | 0.37 | 5.81 | 0.54/Add |
| Dox+ Ber | 16 | 106.66 | 5.33 | 64 | 0.33 | 0.6 | 3 | 0.93/Add |
| Dox+ Thy | 16 | 106.66 | 7.33 | 42.66 | 0.45 | 0.4 | 2.18 | 0.85/Add |
| Dox+ Que | 16 | 298.66 | 5.33 | 90.66 | 0.33 | 0.30 | 3 | 0.63/Add |
| Dox+Gal | 16 | 298.66 | 6.66 | 106.66 | 0.41 | 0.35 | 2.4 | 0.77/Add |
| Cfz+Cur | 42.66 | 149.33 | 18.66 | 32 | 0.43 | 0.21 | 2.28 | 0.65/Add |
| Cfz+Plu | 42.66 | 50.66 | 6.83 | 27.33 | 0.16 | 0.53 | 6.24 | 0.69/Add |
| Cfz+Ber | 42.66 | 170.66 | 11.33 | 53.33 | 0.26 | 0.31 | 3.76 | 0.57/Add |
| Cfz+Thy | 42.66 | 133.3 | 29.33 | 32 | 0.68 | 0.24 | 1.45 | 0.92/Add |
| Cfz+Que | 42.66 | 192 | 18.66 | 74.66 | 0.43 | 0.38 | 2.28 | 0.82/Add |
| Cfz+Gal | 42.66 | 298.66 | 37.33 | 149.33 | 0.87 | 0.5 | 1.14 | 1.37/Ind |
| Amx+Cur | 37.33 | 128 | 8 | 74.66 | 0.21 | 0.58 | 4.66 | 0.79/Add |
| Amx+Plu | 37.33 | 16 | 10.83 | 8 | 0.29 | 0.5 | 3.44 | 0.79/Add |
| Amx+ Ber | 37.33 | 170.66 | 5.33 | 85.33 | 0.14 | 0.5 | 7 | 0.64/Add |
| Amx+Thy | 37.33 | 149.33 | 14 | 64 | 0.37 | 0.42 | 2.66 | 0.80/Add |
| Amx+Que | 37.33 | 256 | 14 | 64 | 0.37 | 0.25 | 2.66 | 0.62/Add |
| Amx+Gal | 37.33 | 298.66 | 23.33 | 224 | 0.62 | 0.75 | 1.6 | 1.37/Add |
| Kan+Cur | 14.66 | 170.66 | 4.33 | 53.33 | 0.29 | 0.31 | 3.38 | 0.60/Add |
| Kan+Plu | 14.66 | 32 | 2.66 | 12.66 | 0.18 | 0.39 | 5.5 | 0.57/Add |
| Kan+Ber | 14.66 | 170.66 | 6.16 | 74.66 | 0.42 | 0.43 | 2.37 | 0.85/Add |
| Kan+Thy | 14.66 | 128 | 8.33 | 128 | 0.56 | 1 | 1.76 | 1.56/Ind |
| Kan+Que | 14.66 | 170.66 | 14.66 | 53.33 | 1 | 0.31 | 1 | 1.31/Ind |
| Kan+Gal | 14.66 | 298.66 | 8 | 106.66 | 0.54 | 0.35 | 1.83 | 0.90/Add |
| Ery+Cur | 44 | 106.66 | 7.33 | 85.33 | 0.16 | 0.8 | 6 | 0.96/Add |
| Ery+Plu | 44 | 13.333 | 9.33 | 4.66 | 0.21 | 0.35 | 4.71 | 0.56/Add |
| Ery+Ber | 44 | 10.66 | 16.33 | 53.33 | 0.37 | 0.5 | 2.69 | 0.87/Add |
| Ery+Thy | 44 | 13.33 | 9.33 | 4.66 | 0.21 | 0.35 | 4.71 | 0.56/Add |
| Ery+Que | 44 | 277.33 | 43.33 | 138.66 | 0.98 | 0.5 | 1.01 | 1.48/Ind |
| Ery+Gal | 44 | 298.66 | 28 | 202.66 | 0.63 | 0.67 | 1.57 | 1.31/Ind |
| Cfx+Plu | 42.66 | 16 | 9.33 | 9.33 | 0.21 | 0.58 | 4.57 | 0.80/Add |
| Cfx+Ber | 42.66 | 170.66 | 8 | 64 | 0.18 | 0.37 | 5.33 | 0.56/Add |
| Cfx+Thy | 42.66 | 149.33 | 34.66 | 106.66 | 0.81 | 0.71 | 1.23 | 1.52/Add |
| Cfx+Que | 42.66 | 384 | 13.33 | 85.33 | 0.31 | 0.22 | 3.2 | 0.53/Add |
| Cfx+Gal | 42.66 | 298.66 | 18.66 | 202.66 | 0.43 | 0.67 | 2.28 | 1.11/Ind |

MBEC: Mean of the minimum biofilm eradication concentrations; FIC: fractional inhibitory concentrations; FICI: fractional inhibitory index (FICI); NPs: natural products; Add: additivity; Ind: indifference; Amk: amikacin; Dox: doxycycline; Cfz: cefazolin; Amx: amoxicillin; Kan: kanamycin; Ery: erythromycin; Cfx: cefixime; Cur: curcumin; Plu: plumbagin; Ber: berberine; Thy: thymol; Que: quercetin; Gal: gallic acid.

**S8: Additive/indifferent interaction of antibiotic and natural product combinations on Staphylococcus saprophyticus biofilm eradication.**

| Antimicrobial agent in combination (*S. saprophyticus*) | MBEC (µg/mL) | | | |  | |  |  |
| --- | --- | --- | --- | --- | --- | --- | --- | --- |
|  | Alone | | Combined | | FIC | | MIC reduction fold of ATB | FICI/Interpretation |
|  | ATB | NPs | ATB | NPs | ATB | NPs |  |  |
| Amk+ Cur | 13.33 | 170.66 | 8 | 64 | 0.6 | 0.37 | 1.66 | 0.97/Add |
| Amk+Plu | 13.33 | 10.66 | 5.33 | 2.66 | 0.4 | 0.25 | 2.5 | 0.65/Add |
| Amk+Ber | 13.33 | 170.66 | 6.66 | 37.33 | 0.5 | 0.21 | 2 | 0.71/Add |
| Amk+ Thy | 13.33 | 106.66 | 6 | 48 | 0.45 | 0.45 | 2.22 | 0.9/Add |
| Amk+Que | 13.33 | 170.66 | 5.33 | 64 | 0.4 | 0.37 | 2.5 | 0.77/Add |
| Amk+Gal | 13.33 | 298.66 | 9.33 | 85.33 | 0.7 | 0.28 | 1.42 | 0.98/Add |
| Dox+Cur | 9.33 | 170.66 | 3.33 | 85.33 | 0.35 | 0.5 | 2.8 | 0.85/Add |
| Dox+Plu | 9.33 | 10.66 | 2.66 | 13.33 | 0.28 | 1.25 | 3.5 | 1.53/Ind |
| Dox+ Ber | 9.33 | 128 | 4.66 | 21.33 | 0.5 | 0.16 | 2 | 0.66/Add |
| Dox+ Thy | 9.33 | 106.66 | 4.66 | 53.33 | 0.5 | 0.5 | 2 | 1/Ind |
| Dox+ Que | 9.33 | 298.66 | 3.66 | 69.33 | 0.39 | 0.23 | 2.54 | 0.62/Add |
| Dox+Gal | 9.33 | 128 | 4.66 | 21.33 | 0.5 | 0.16 | 2 | 0.66/Add |
| Cfz+Cur | 10.66 | 170.66 | 4.66 | 37.33 | 0.43 | 0.21 | 2.28 | 0.65/Add |
| Cfz+Plu | 10.66 | 10.66 | 4 | 14.66 | 0.37 | 1.37 | 2.66 | 1.75/Ind |
| Cfz+Ber | 10.66 | 85.33 | 4.66 | 42.66 | 0.43 | 0.5 | 2.28 | 0.93/Add |
| Cfz+Thy | 10.66 | 85.33 | 5.33 | 48 | 0.5 | 0.56 | 2 | 1.06/Ind |
| Cfz+Que | 10.66 | 426.66 | 8 | 53.33 | 0.75 | 0.12 | 1.33 | 0.87/Add |
| Cfz+Gal | 10.66 | 426.66 | 4.66 | 234.66 | 0.43 | 0.55 | 2.28 | 0.98/Add |
| Amx+Cur | 9.33 | 213.33 | 4 | 42.66 | 0.42 | 0.2 | 2.33 | 0.62/Add |
| Amx+Plu | 9.33 | 106.6 | 3.33 | 9.33 | 0.35 | 0.87 | 2.8 | 1.23/Ind |
| Amx+Ber | 9.33 | 128 | 5.33 | 64 | 0.57 | 0.5 | 1.75 | 1.07/Ind |
| Amx+Thy | 9.33 | 234.66 | 5.33 | 53.33 | 0.57 | 0.22 | 1.75 | 0.79/Add |
| Amx+Quer | 9.33 | 256 | 5.33 | 85.33 | 0.57 | 0.33 | 1.75 | 0.90/Add |
| Amx+Gal | 9.33 | 213.33 | 6.66 | 170.66 | 0.71 | 0.8 | 1.4 | 1.51/Ind |
| Kan+Cur | 10.66 | 170.66 | 3.33 | 53.33 | 0.31 | 0.31 | 3.2 | 0.62/Add |
| Kan+Plu | 10.66 | 10.66 | 4.66 | 4.66 | 0.43 | 0.43 | 2.28 | 0.87/Add |
| Kan+Ber | 10.66 | 128 | 3.33 | 37.33 | 0.31 | 0.29 | 3.2 | 0.60/Add |
| Kan+Thy | 10.66 | 106.66 | 2.75 | 37.33 | 0.25 | 0.35 | 3.87 | 0.60/Add |
| Kan+Que | 10.66 | 170.66 | 8 | 69.33 | 0.75 | 0.40 | 1.33 | 1.15/Ind |
| Kan+Gal | 10.66 | 298.66 | 4.66 | 234.66 | 0.43 | 0.78 | 2.28 | 1.22/Ind |
| Ery+Cur | 29.33 | 170.66 | 8 | 42.66 | 0.27 | 0.25 | 3.66 | 0.52/Add |
| Ery+Plu | 29.33 | 10.66 | 26.66 | 4.66 | 0.90 | 0.43 | 1.1 | 1.34/ Ind |
| Ery+Thy | 29.33 | 106.66 | 11.33 | 37.33 | 0.38 | 0.35 | 2.58 | 0.73/Add |
| Ery+Que | 29.33 | 256 | 9.33 | 64 | 0.31 | 0.25 | 3.14 | 0.56/Add |
| Ery+Gal | 29.33 | 341.33 | 25.33 | 298.66 | 0.86 | 0.87 | 1.15 | 1.73/Ind |
| Cfx+Cur | 48 | 170.66 | 16 | 53.33 | 0.33 | 0.31 | 3 | 0.64/Add |
| Cfx+Ber | 48 | 106.66 | 12 | 69.33 | 0.25 | 0.65 | 4 | 0.9/Add |
| Cfx+Que | 48 | 298.66 | 32 | 256 | 0.66 | 0.85 | 1.5 | 1.52/Ind |
| Cfx+Gal | 48 | 298.66 | 34.66 | 64 | 0.72 | 0.21 | 1.38 | 0.93/Add |

MBEC: Mean of the minimum eradication concentrations; FIC: fractional inhibitory concentrations; FICI: fractional inhibitory index (FICI); NPs: natural products; Syn: synergistic; Add: additivity; Ind: indifference; Amk: amikacin; Dox: doxycycline; Cfz: cefazolin; Amx: amoxicillin; Kan: kanamycin; Ery: erythromycin; Cfx: cefixime; Cur: curcumin; Plu: plumbagin; Ber: berberine; Thy: thymol; Que: quercetin; Gal: gallic acid.

**S9**: **Additive/indifferent interaction of antibiotic and natural product combinations on Staphylococcus epidermidis biofilm eradication.**

| **Antimicrobial agent in combination (*S. epidermidis*)** | **MBEC (µg/mL)** | | | |  | |  |  |
| --- | --- | --- | --- | --- | --- | --- | --- | --- |
|  | **Alone** | | **Combined** | | **FIC** | | **MIC reduction fold of ATB** | **FICI/Interpretation** |
|  | **ATB** | **NPs** | **ATB** | **NPs** | **ATB** | **NPs** |  |  |
| Amk+ Cur | 21.33 | 106.66 | 9.33 | 42,66 | 0.43 | 0.4 | 2.28 | 0.83/Add |
| Amk+Plu | 21.33 | 10.66 | 7.33 | 8 | 0.34 | 0.75 | 2.90 | 1.09/Ind |
| Amk+Ber | 21.33 | 170.66 | 5.33 | 53.33 | 0.25 | 0.31 | 4 | 0.56/Add |
| Amk+ Thy | 21.33 | 106.66 | 6.66 | 53.33 | 0.31 | 0.5 | 3.2 | 0.81/Add |
| Amk+Que | 21.33 | 298.66 | 16 | 256 | 0.75 | 0.85 | 1.33 | 1.60/Ind |
| Amk+Gal | 21.33 | 266.66 | 9.33 | 34.66 | 0.43 | 0.13 | 2.28 | 0.56/Add |
| Dox+Cur | 16 | 106.66 | 6 | 48 | 0.37 | 0.45 | 2.66 | 0.82/Add |
| Dox+ Ber | 16 | 170.66 | 5.33 | 69.33 | 0.33 | 0.40 | 3 | 0.73/Add |
| Dox+ Thy | 16 | 128 | 4.66 | 53.33 | 0.29 | 0.41 | 3.42 | 0.70/Add |
| Dox+ Que | 16 | 138.66 | 10.66 | 37.33 | 0.66 | 0.26 | 1.5 | 0.93/Add |
| Dox+Gal | 16 | 298.66 | 9.33 | 96 | 0.58 | 0.32 | 1.71 | 0.90/Add |
| Cfz+Cur | 10.66 | 106.66 | 4.66 | 69.33 | 0.43 | 0.65 | 2.28 | 1.08/Ind |
| Cfz+Plu | 10.66 | 53.33 | 4.66 | 4.33 | 0.43 | 0.08 | 2.28 | 0.51/Add |
| Cfz+Ber | 10.66 | 170.66 | 4.66 | 42.66 | 0.43 | 0.25 | 2.28 | 0.68/Add |
| Cfz+Thy | 10.66 | 128 | 4 | 58.66 | 0.37 | 0.45 | 2.66 | 0.83/Add |
| Cfz+Que | 10.66 | 170.66 | 8 | 128 | 0.75 | 0.75 | 1.33 | 1.5/Ind |
| Cfz+Gal | 10.66 | 426.66 | 9.33 | 213.33 | 0.87 | 0.5 | 1.14 | 1.37/Ind |
| Amx+Cur | 16 | 106.66 | 8 | 69.33 | 0.5 | 0.65 | 2 | 1.15/Ind |
| Amx+Plu | 16 | 13.33 | 6.66 | 4.66 | 0.41 | 0.35 | 2.4 | 0.76/Add |
| Amx+ Ber | 16 | 149.33 | 6.66 | 37.33 | 0.41 | 0.25 | 2.4 | 0.66/Add |
| Amx+Thy | 16 | 128 | 6.66 | 42.66 | 0.41 | 0.33 | 2.4 | 0.75/Add |
| Amx+Que | 16 | 298.66 | 12 | 170.66 | 0.75 | 0.57 | 1.33 | 1.32/Ind |
| Amx+Gal | 16 | 426.66 | 9.33 | 224 | 0.58 | 0.52 | 1.71 | 1.10/Ind |
| Kan+Cur | 13.33 | 106.66 | 6.66 | 58.66 | 0,5 | 0.55 | 2 | 1.05/Ind |
| Kan+Plu | 13.33 | 29.33 | 6.83 | 6.66 | 0,51 | 0.22 | 1.95 | 0.73/Add |
| Kan+Ber | 13.33 | 213.33 | 4 | 48 | 0,3 | 0.22 | 3.33 | 0.52/Add |
| Kan+Thy | 13.33 | 106.66 | 3.33 | 45.33 | 0,25 | 0.42 | 4 | 0.67/Add |
| Kan+Que | 13.33 | 298.66 | 10.66 | 213.33 | 0,8 | 0.71 | 1.25 | 1.51/Ind |
| Kan+Gal | 13.33 | 426.66 | 8 | 266.66 | 0,6 | 0.62 | 1.66 | 1.22/Ind |
| Ery+Cur | 18.66 | 128 | 8 | 42.66 | 0,42 | 0.33 | 2.33 | 0.76/Add |
| Ery+Plu | 18.66 | 10.66 | 8.66 | 5.33 | 0,46 | 0.5 | 2.15 | 0.96/Add |
| Ery+Ber | 18.66 | 213.33 | 4.66 | 74.66 | 0,25 | 0.35 | 4 | 0.6/Add |
| Ery+Thy | 18.66 | 128 | 8.33 | 69.33 | 0,44 | 0.54 | 2.24 | 0.98/Add |
| Ery+Que | 18.66 | 213.33 | 13.33 | 69.33 | 0,71 | 0.32 | 1.4 | 1.03/Add |
| Ery+Gal | 18.66 | 426.66 | 6 | 106.66 | 0,32 | 0.25 | 3.11 | 0.57/Add |
| Cfx+Cur | 42.66 | 106.66 | 24 | 42.66 | 0,56 | 0.4 | 1.77 | 0.96/Add |
| Cfx+Ber | 42.66 | 170.66 | 18.66 | 32 | 0,43 | 0.18 | 2.28 | 0.62/Add |
| Cfx+Thy | 42.66 | 106.66 | 13.33 | 53.33 | 0,31 | 0.5 | 3.2 | 0.81/Add |
| Cfx+Quer | 42.66 | 170.66 | 16 | 64 | 0,37 | 0.37 | 2.66 | 0.75/Add |
| Cfx+Gal | 42.66 | 426.66 | 42.66 | 341.33 | 1 | 0.8 | 1 | 1.8/Ind |

MBEC: mean of the minimum eradication concentrations; FIC: fractional inhibitory concentrations; FICI: fractional inhibitory index (FICI); NPs: natural product; Add: additivity; Ind: indifference; Amk: amikacin; Dox: doxycycline; Cfz: cefazolin; Amx: amoxicillin; Kan: kanamycin; Ery: erythromycin; Cfx: cefixime; Cur: curcumin; Plu: plumbagin; Ber: berberine; Thy: thymol; Que: quercetin; Gal: gallic acid.
